# Targeted Next-Generation Sequencing and Informatics as an Effective Tool to Establish the Composition of Bovine Piroplasm Populations in Endemic Regions

**DOI:** 10.3390/microorganisms9010021

**Published:** 2020-12-23

**Authors:** Abdul Ghafar, Anson V. Koehler, Ross S. Hall, Charles G. Gauci, Robin B. Gasser, Abdul Jabbar

**Affiliations:** Department of Veterinary Biosciences, Melbourne Veterinary School, Faculty of Veterinary and Agricultural Sciences, The University of Melbourne, Werribee, VIC 3030, Australia; abdul.ghafar@unimelb.edu.au (A.G.); anson.koehler@unimelb.edu.au (A.V.K.); rossh@unimelb.edu.au (R.S.H.); charlesg@unimelb.edu.au (C.G.G.); robinbg@unimelb.edu.au (R.B.G.)

**Keywords:** targeted next-generation sequencing, informatics, protist populations, *Babesia*, *Theileria*, bovines, 18S ribosomal RNA

## Abstract

Protists of the genera *Babesia* and *Theileria* (piroplasms) cause some of the most prevalent and debilitating diseases for bovines worldwide. In this study, we established and used a next-generation sequencing-informatic approach to explore the composition of *Babesia* and *Theileria* populations in cattle and water buffalo in a country (Pakistan) endemic for these pathogens. We collected individual blood samples from cattle (*n* = 212) and water buffalo (*n* = 154), extracted genomic DNAs, PCR-amplified the V4 hypervariable region of 18S small subunit rRNA gene from piroplasms, sequenced amplicons using Illumina technology, and then analysed data using bioinformatic platforms. The results revealed piroplasms in 68.9% (252/366) samples, with overall occurrence being markedly higher in cattle (85.8%) than in water buffaloes (45.5%). *Babesia* (*B.*) *occultans* and *Theileria* (*T.*) *lestoquardi*-like species were recorded for the first time in Pakistan, and, overall, *T. annulata* was most commonly detected (65.8%) followed by *B. bovis* (7.1%), *B. bigemina* (4.4%), and *T. orientalis* (0.5%), with the genetic variability within *B. bovis* being pronounced. The occurrence and composition of piroplasm species varied markedly across different agro-ecological zones. The high detection of *T. annulata* in asymptomatic animals suggested a relatively high level of endemic stability of tropical theileriosis in the bovine population.

## 1. Introduction

Tick-borne diseases of ruminants [e.g., cattle (*Bos indicus* and *Bos taurus*) and buffalo (*Bubalus bubalis*)]—including piroplasmosis (i.e., babesiosis and theileriosis)—significantly affect the productivity of livestock globally [1,2], leading to a major adverse impact on food supply and the economy, particularly in Africa, the Americas, Asia, and Australia [3]. Bovine babesiosis is the most important tick-borne disease of cattle, and is caused by *Babesia* (*B.*) *bovis* and *B. bigemina* (in tropical and sub-tropical regions) as well as *B. divergens* (in the temperate areas) [4,5,6]. *Babesia bovis* and *B. bigemina* are mainly transmitted by *Rhipicephalus* (*Boophilus*) *microplus* and *R.* (*B.*) *annulatus*, and *B. divergens* by *Ixodes ricinus* [5,6]. Although vertical transmission is uncommon for *Babesia* spp., it has been described for *B. divergens* [7]. The clinical manifestation of babesiosis includes anorexia, lethargy, anaemia, icterus, tachycardia, and/or death due to respiratory distress [8,9]. *Theileria parva* causes bovine theileriosis (known as East Coast Fever, transmitted by *R. appendiculatus*) in Eastern, Central, and Southern Africa, and *T. annulata* causes tropical theileriosis (transmitted by *Hyalomma* spp.) in Southern Europe, Asia, and Africa [10]. The clinical manifestation of tropical theileriosis includes anaemia, hypoxia, enlarged superficial lymph nodes, fever, respiratory distress, and/or death [11]. If animals recover from acute babesiosis and theileriosis, they assume a carrier status (with premunition), in which they maintain long-term subclinical infection with piroplasms, which can be ingested by female ticks as they take their blood meal from the animal [12].

Subclinical piroplasm infections are usually not reliably diagnosed by conventional microscopic examination of Giemsa-stained thin and thick blood smears [13] or serological tests, such as the enzyme-linked immunosorbent assay (ELISA) and the indirect fluorescent antibody test (IFAT) [13,14], due to limited sensitivity or specificity. Although conventional (singleplex) PCRs targeting the 18S nuclear ribosomal RNA gene [15] offers suitable analytical sensitivity and specificity [16], most are not able to accurately discern multiple species in a single sample [17]. Although multiplex PCR assays can overcome this limitation, they can be challenging to establish and standardise. On the other hand, next-generation (deep) sequencing (NGS) of amplicons produced by PCR from informative target genes using relatively ‘conserved’ primers allows the identification of multiple taxa or species within a sample with high specificity and sensitivity, as well as the assessment of genetic variation and their relative abundance [18,19,20,21]. The hypervariable V4 region of the nuclear 18S rRNA gene is reported to be a suitable target for the identification/classification of piroplasm and the study of population structures [20,22,23,24,25,26,27].

Given that ticks and tick-borne diseases are a major constraint to the livestock industry and agriculture in Pakistan [28], there have been some epidemiological investigations of piroplasms of bovines using conventional microscopic methods [29,30,31,32,33,34,35,36,37]. However, there has been no detailed genetic investigation of piroplasm populations in bovines or their ticks in this country. In the present study, we established and employed PCR-based NGS of the V4 region of 18S rRNA to elucidate the composition of piroplasm populations in cattle and buffaloes in distinct geographical regions of Pakistan known to be endemic for these protists.

## 2. Materials and Methods

### 2.1. Collection of Blood Samples and DNA Extraction

**Figure 1:**
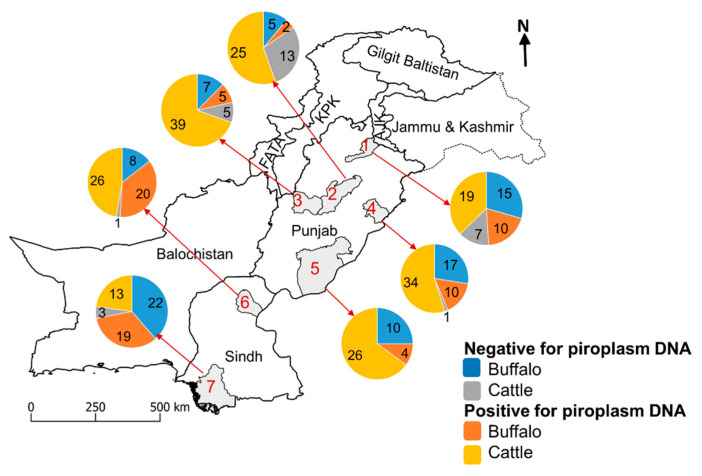
Map of Pakistan showing the numbers of cattle and buffaloes in which piroplasm species were detected in Jhelum (1), Jhang (2), Layyah (3), Okara (4), Bahawalpur (5), Sukkur (6), and Thatta (7) districts of Punjab and Sindh provinces. Abbreviation: FATA, Federally Administered Tribal Area; KPK, Khyber Pakhtunkhwa.

The collection of bovine blood samples was approved by the Animal Ethics Committee at the Faculty of Veterinary and Agricultural Sciences, The University of Melbourne (permit no. 714216). From September to November 2017, blood samples (*n* = 366) were collected from the bovine (i.e., cattle and buffalo) populations, with no history of clinical piroplasmosis, located in 25 villages in the districts Bahawalpur, Jhang, Jhelum, Layyah, and Okara of Punjab and 10 villages in districts Sukkur and Thatta of Sindh province (Figure 1). These villages are located in five agro-ecological zones of Pakistan, which differ in climate, land use, and soil type, and the majority of bovine population of Pakistan occurs in these zones [38,39]. Small-holder farms (with ≤ 10 bovines) from each village were selected because they harbour > 90% of the bovine population in the country [39]. Blood samples were collected from different age-groups (< 1 year, 1–3 years, and >3 years) of cattle (*n* = 212; male = 38; female = 174) and buffaloes (*n* = 154; male = 39; female = 115) via venipuncture using syringes (BD, Franklin Lakes, NJ, USA) into anticoagulant (EDTA)-coated vacutainers (BD, Franklin Lakes, NJ, USA). All samples were stored at −20 °C prior to further processing.

From aliquots (100 μL) of individual blood samples, genomic DNAs were extracted using the DNeasy Blood & Tissue Kit (Qiagen, Hilden, Germany), according to the manufacturer’s recommendations, and were stored at −20 °C until further use.

### 2.2. PCR-Based Next-Generation Sequencing

The 18S rRNA hyper-variable region V4 was PCR-amplified from individual genomic DNA samples. Primary PCR was achieved using primers RLBF and RLBR [15] bearing 5′-adaptor sequences (F: 5′-GTGACCTATGAACTCAGGAGTC-primer-3′; R: 5′-CTGAGACTTGCACATCGC-AGC-primer-3′). This PCR was carried out in a volume of 25 µL, containing 3.12 mM of each deoxynucleotide triphosphate (dNTP), 6.25 pmol of each adaptor primer, 125 mM MgCl_2_, 5X GoTaq reaction buffer, and 0.6 U of GoTaq flexi DNA polymerase (Promega, Madison, WI, USA). The thermocycling conditions consisted of an initial denaturation at 95 °C for 5 min followed by 18 cycles of 95 °C for 15 s, 52 °C for 20 s, and 72 °C for 30 s followed by final elongation at 72 °C for 7 min. Positive (*B. bovis* and *T. annulata*) and negative (template-free and DNA of haemoparasites-free bovine blood) controls were included in each PCR. A subset of samples (10%) was randomly selected using the “sample()” command in R version 4.0.3 [40] and used as technical replicates. Amplicons were purified using 1X Ampure Beads (Beckman Coulter, Brea, CA, USA).

Secondary PCR was carried out to introduce 8-base forward and reverse indices (Illumina, San Diego, CA, USA) into individual amplicons (i.e., multiplexing) for subsequent sequencing of all amplicons in a single run. Twenty-three forward and 29 reverse indices were used, allowing multiplexing of 418 amplicons (i.e., 366 samples, 37 replicates, and 15 controls). Triplicates of each of the confirmed positive controls (*B. bovis* and *T. annulata*) and negative control samples [DNAs from haemoparasites-free bovine-bloods, and no-DNA i.e., Ambion nuclease-free water (Life Technologies, Austin, TX, USA) used in adaptor PCR as well as in indexing PCR] were used to exclude cross-contamination. Aliquots (10 µL) of each purified amplicon from the first PCR was used as a template along with 10 μL of OneTaq^®^ 2X Master Mix (New England Biolabs, Ipswich, MA, USA) and 0.5 μL of each index. Thermocycling conditions were an initial denaturation at 95 °C for 3 min followed by 24 cycles of 95 °C for 15 s, 60 °C for 30 s, and 72 °C for 30 s, and a final extension at 72 °C for 7 min.

Following an assessment of the quality of amplicons using an Agilent 2200 TapeStation (Agilent Technologies, Santa Clara, CA, USA), amplicons were purified using 1X Ampure Beads (Beckman Coulter, Brea, CA, USA). Then, aliquots (5 μL) of 418 individual, purified amplicons were pooled and sequenced using a 600-cycle (2 × 300 bp paired-end reads) kit (MiSeq Reagent Kit v3, Illumina San Diego, CA, USA) on a MiSeq platform (Illumina, San Diego, CA, USA).

An evaluation of this nested PCR-based NGS method revealed that the primary and secondary PCRs could reproducibly amplify ≤ 1.0 picogram/µL from pure genomic DNA of *B. bovis* or *B. bigemina*, thereby indicating high analytical sensitivity.

### 2.3. Pre-Processing and Analysis of Sequence Data, and Taxonomic Assignment

The demultiplexing of raw data was carried out using in-house software, resulting in FASTQ sequence files. The paired-end FASTQ files were uploaded into a QIIME2 v. 2019.10 [41] environment for further processing and informatic analyses. Adaptor, primer, and index sequences were trimmed from all forward and reverse reads using cutadapt plugin [42]. Trimmed reads were then imported as QIIME2 compatible “.qza” files. Quality plots were generated for randomly selected 10,000 sequences each from forward and reverse reads separately to assess the read-quality in q2view. This allowed the selection of truncation parameters to remove reads of low quality (≤30).

The DADA2 plugin [43] was used to remove chimeras and low-quality reads and to dereplicate and merge paired-end reads. This resulted in ASVs instead of operational taxonomic units (OTU), thus allowing a comparatively accurate measure of diversity [44]. The shorter length reads (<410 bp) and samples with less than a total of 10 reads (*n* = 11) were excluded from further analyses cf. [45]. The SILVA SSU 138 reference database was downloaded (http://www.arb-silva.de/download/arb-files) and all unpublished *Babesia* spp. and *Theileria* spp. data removed. The q-2 feature-classifier was used to train and test the curated SILVA database. The curated database was used to classify the ASVs to species/taxa through the scikit-learn classifier [46]. Subsets of ASVs were selected for each species/taxon and their taxonomic assignment verified using BLASTn (NCBI). A taxonomic bar-plot of ASVs was generated using the QIIME2 taxa barplot visualiser. Alpha rarefaction plots were generated using MAFFT [47] and FastTree [48] plugins in QIIME2 to ensure sufficient sequencing depth.

### 2.4. Phylogenetic Analyses

Unique ASVs determined herein were aligned using MUSCLE v.3.8.31 [49] within MEGA 7.0 using default settings [50]. Pairwise comparisons of aligned ASVs were performed to calculate nucleotide differences using BioEdit version X [51]. Subsequently, published, matching reference sequences of *Babesia* and *Theileria* spp. from bovines were retrieved from GenBank and aligned with respective piroplasm sequences identified in this study. Alignments were performed using MUSCLE in MEGA using default settings and were then trimmed to uniform lengths of 434 (*Babesia* spp.) or 459 (*Theileria* spp.) nucleotides (Figures S1 and S2).

The evolutionary models for individual DNA sequence alignments were determined using the Akaike information criterion test in jModelTest v.3.7 [52]. NJ and ML trees were constructed based on the Tamura-Nei method [53] with gamma-distributed (shape parameter for *Babesia* spp. = 0.46 and *Theileria* spp. = 0.693) site variation and complete deletion using MEGA. The bootstrapping method (10,000 replicates) was used to infer the reliability of internal branches [54]. *Babesia microti* (GenBank accession no. AY918951) and *T. ornithorhynchi* (KT937390) were used as outgroups. The phylogenetic trees built using the ML and NJ methods were created using the software FigTree 1.4.3 (http://tree.bio.ed.ac.uk/software/figtree/), and their topologies were compared.

### 2.5. Statistical Analyses

Data were analysed using GraphPad Prism 5 program (GraphPad Software Inc., La Jolla, CA, USA) and R version 3.4.3 [40]. Figure images were produced using Microsoft Excel for Office 365 and ggplot2 [55], phyloseq [56], and/or UpSetR [57] packages in R.

### 2.6. Data Availability Statement

The NGS data of 18S rRNA produced in the present study are available from the GenBank under accession numbers: MW165561-MW165706 (*Theileria annulata*), MW165707-MW165710 (*T. orientalis*), MW165716 (*T. lestoquardi*-like), MW165717-MW165730 (*Babesia bovis*), MW165731-MW165739 (*B. bigemina*), and MW165740 (*B. occultans*).

## 3. Results

### 3.1. Sequence Data Sets and Definition of ASVs

Sequence data were obtained for 252 of the 366 samples analysed. In total, 3,876,271 curated sequence reads represented these 252 samples (182 from cattle and 70 from buffalo), and 175 amplicon sequence variants (ASVs) (mean length: 453 bp; standard deviation = 13.4) were recorded (Figures S1 and S2). More ASVs were recorded for *Theileria* spp. (*T. annulata* = 146, *T. orientalis* = 4, and *T. lestoquardi*-like = 1) than for *Babesia* spp. (*B. bovis* = 14, *B. bigemina* = 9, and *B. occultans* = 1) (Figures S3 and S4).

Within individual species, pairwise comparison of ASVs (Tables S1 and S2) showed the highest level of variation for *B. bovis* (0.3–6.3%), followed by *T. orientalis* (0.3–1.8%), *B. bigemina* (0.3–1.4%) and *T. annulata* (0.3–0.9%). For *Babesia* spp., sequence differences were highest (21.6–23%) between *B. bovis* and *B. bigemina*, followed by 20.2–21.1% between *B. bovis* and *B. occultans* and 7.7–8.1% between *B. bigemina* and *B. occultans*. For *Theileria*, differences of 7.7–8.1% were recorded between *T. lestoquardi*-like and *T. orientalis*, 5.3–7% between *T. annulata* and *T. orientalis*, and 2.2–2.9% between *T. annulata* and *T. lestoquardi*-like.

### 3.2. Phylogenetic Relationships of ASVs

**Figure 2:**
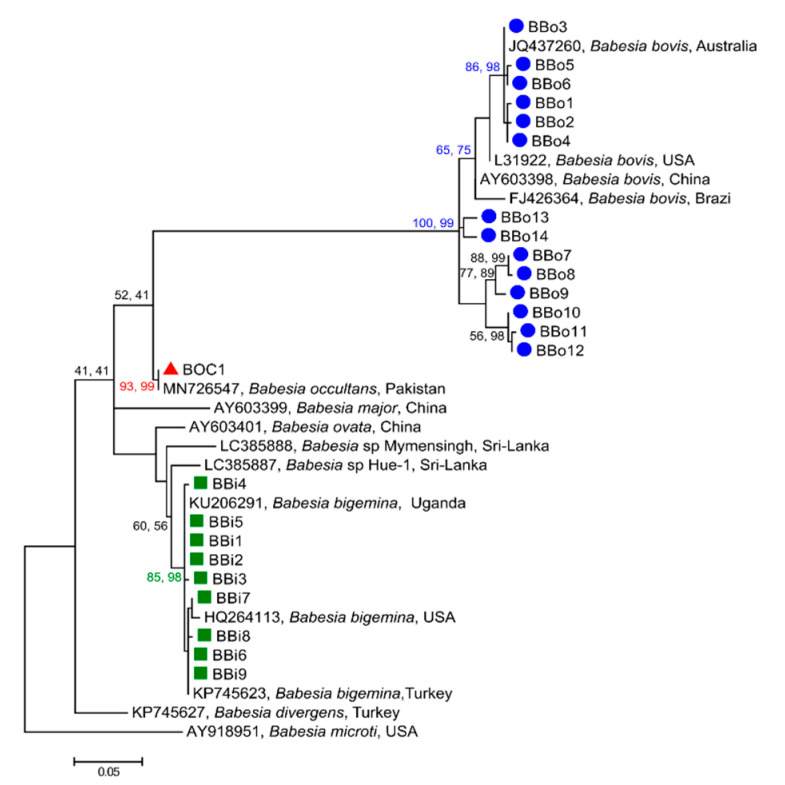
Phylogenetic relationships of amplicon sequence variants (ASVs) of Babesia species determined in this study (coloured symbols) with reference sequences from previous studies. The relationships were inferred based on separate analyses of 18S rRNA sequence data (aligned over 386 bp) using the Maximum Likelihood (ML) and Neighbor-Joining (NJ) methods. Babesia microti (AY918951) was used as outgroup. Nodal support values are indicated as bootstrap values for the NJ (first) and ML (second) method. The scale bar indicates distance.

Phylogenetic analyses of 24 ASVs representing the genus *Babesia* identified three distinct species (Figure 2). As the trees obtained by Maximum Likelihood (ML) and Neighbour Joining (NJ) analyses of ASV data had a very similar topology, only ML trees are presented. Clade 1 contained 14 sequences (BBo1-BBo14 represented as blue circles in Figure 2), which grouped with 18S sequences of *B. bovis* from Australia (GenBank JQ437260), Brazil (GenBank FJ426364), China (GenBank AY603398), and the USA (GenBank L31922), with strong statistical support (bootstrap values of NJ = 100% and ML = 99%). The second clade comprised of one ASV (BOC1 represented as red triangle in Figure 2) which clustered with a reference sequence of *B. occultans* from Pakistan (GenBank MN726547), with moderate to strong nodal support (NJ = 93% and ML = 99%). Clade 3 consisted nine sequences (BBi1-Bbi9 represented as green squares in Figure 2) which grouped with 18S sequences of *B. bigemina* previously published from Turkey (GenBank KP745623), the USA (GenBank HQ264113), and Uganda (GenBank KU206291), with medium to strong nodal support (NJ = 85% and ML = 98%).

**Figure 3:**
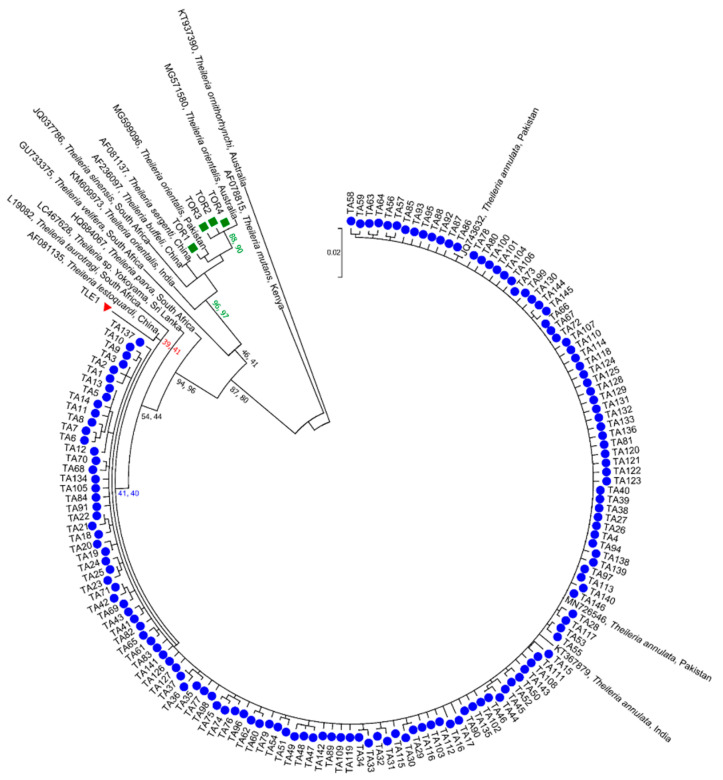
Phylogenetic relationships of amplicon sequence variants (ASVs) of *Theileria* species determined in this study (coloured symbols) with reference sequences from previous studies. The relationships were inferred based on separate analyses of 18S rRNA sequence data (aligned over 386 bp) using the Maximum Likelihood (ML) and Neighbor-Joining (NJ) methods. *Theileria ornithorhynchi* (KT937390) was used as an outgroup. Nodal support values are indicated as bootstrap values for the NJ (first) and ML (second) method. The scale bar indicates distance.

Phylogenetic analyses of 151 ASVs representing the genus *Theileria* revealed three clades (Figure 3). The majority of the ASVs in clade 1 (TA1-TA146 represented as blue circles in Figure 3) grouped with reference sequences of *T. annulata* from Pakistan (GenBank JQ743632 and MN726546) and India (GenBank KY367879), with low statistical support (NJ = 41% and ML = 40%). Clade 2 contained one ASV (TLE1 represented as red triangle in Figure 3) that clustered with an 18S sequence of *T. lestoquardi* from China (GenBank AF081135), with weak nodal support (NJ = 39% and ML = 41%). The third clade comprised four sequences (TOR1-TOR4 represented as green squares in Figure 3) which grouped with reference sequences of *T. orientalis* complex from Australia (GenBank MG571580), China (GenBank AF236097 and AF081137) and Pakistan (GenBank MG599096), with strong nodal support (NJ = 96% and ML = 97%).

### 3.3. Composition of Piroplasm Populations in Individual Bovines

**Figure 4:**
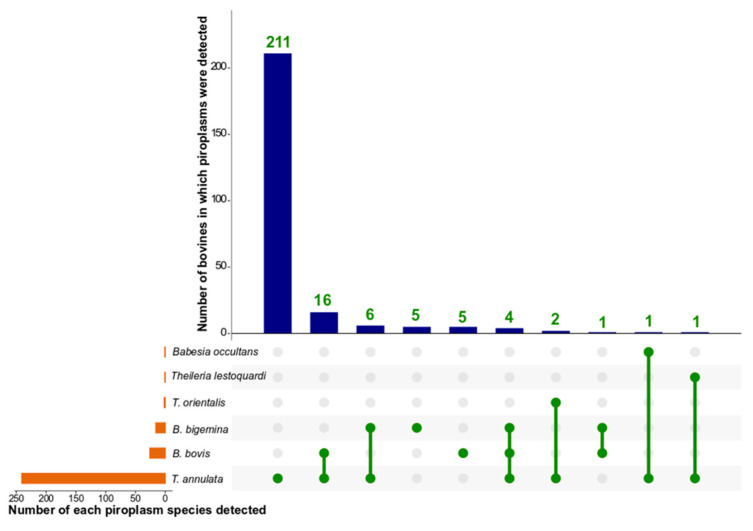
An UpSetR plot of *Babesia* and *Theileria* species detected in the tested bovine population in Pakistan by using PCR-based next-generation (deep) sequencing (NGS). The orange horizontal coordinate columns represent the number of samples which were test-positive for each piroplasm species. The blue vertical columns show the numbers of bovines that are positive for single or coinfections of piroplasm species. The green lines connecting green dots indicate coinfecting piroplasm species.

In total, six piroplasm species belonging to two genera—*Babesia* and *Theileria*—were detected using PCR-based NGS. One to six species of piroplasm were detected in 252 (68.9%) of all 366 samples (animals) tested (Table 1). *Theileria* annulata was detected in most (65.8%) of the 366 animals from all districts, followed by *B. bovis* (7.1%) and *B. bigemina* (4.4%) from six and four districts, respectively. *Theileria* orientalis (0.5%) was detected only in cattle from two districts (Jhang and Okara), *T. lestoquardi*-like (0.3%) was detected in a buffalo from the Thatta district of Sindh and *B. occultans* (0.3%, 1/366) in a cow from Layyah (Table 1). Multiple species of piroplasm were detected in 8.5% (31/366) of bovines studied, with two species in 27 (10.7%) and three in four (1.6%) of infected animals (Figure 4). Specifically, *B. bovis* and *T. annulata* were detected in 16 bovines, *B. bigemina* and *T. annulata* were detected in six, and *T. annulata* and *T. orientalis* were detected in two bovines. *B. bigemina*, *B. bovis*, and *T. annulata* were detected in four animals (Figure 4). Coinfections with *B. bigemina* and *B. bovis*, *T. annulata* and *T. lestoquardi*-like, or *T. annulata* and *B. occultans* were detected in individual animals (Figure 4). Overall, more cattle (n = 18) harboured multiple species of piroplasm than buffaloes (n = 13).

## 4. Discussions

The livestock sector in Pakistan is the mainstay of the agriculture-based economy and contributes 60.6% of its agricultural share of the gross domestic product [58]. Within this context, ~50 million cattle (Bos indicus and Bos taurus) and 41 million water buffaloes (Bubalus bubalis) produce ~97% of the gross milk production in this country [58]. Given that ticks and tick-borne diseases have a major adverse impact on the livestock industry and the dearth of detailed knowledge and understanding of the epidemiology of such diseases in this country [28], there was significant merit in gaining an insight into the abundance and genetic composition of piroplasm populations in the general bovine populations in key farming areas of Pakistan.

The present study showed that piroplasm species are abundant, as they were detected in 68.9% of 366 individuals representing the general bovine population from five zones. *Theileria* annulata was the most commonly detected (95.6%, 241/252), and *B. occultans* and *T. lestoquardi*-like were detected for the first time in bovines in this country. The genetic diversity detected within some piroplasm was conspicuous, and exemplified the utility of PCR-directed NGS to identify existing and novel piroplasm taxa of veterinary and/or public health significance. The variation in composition of piroplasm populations in bovines between or among some districts is likely associated with differing prevalences of suitable tick vectors, as indicated recently [59,60], and encourages detailed investigations of the abundance and diversity of pathogens in vector populations.

*Theileria* annulata—the most abundant piroplasm identified—is the major cause of tropical theileriosis in the region [28]. Tropical theileriosis is one of the most serious constraints on livestock establishments [1,61]. Despite the high abundance of the vector tick, H. anatolicum, in Pakistan [59,60], the abundance of *T. annulata* was surprising because blood samples were collected from asymptomatic animals during the season when the temperature and prevalence of ticks are usually low [62] and because the indigenous cattle (Bo. indicus) and buffaloes (Bu. bubalis) studied are both naturally resistant to TBPs [63,64,65]. Although previous studies have reported a high prevalence of *T. annulata* in clinically healthy animals in Pakistan (30–59.8%) [34,36], Spain (22.4%) [66], and Turkey (39.3%) [67], the occurrence estimated herein exceeds most of the previous estimates, which may, at least in part, relate to the high analytical sensitivity of PCR-directed NGS [68,69].

*Theileria* orientalis—the cause of a “benign” theileriosis—was detected in two cattle from Okara and Jhang districts; this taxon has caused significant outbreaks in countries including Australia, New Zealand, Japan, and the USA [70]. Pathogenic genotypes of *T. orientalis* have been reported in mammalian and tick hosts in countries of the Asia Pacific, including India [71], Sri Lanka [72], China [73], and Pakistan [34,74]. Some evidence indicates that this species was introduced through the import of infected animals from Australia [74], and we think that future work might focus on assessing the molecular epidemiology of oriental theileriosis in cattle (Bo. taurus) imported to Pakistan from Australia and the USA, as the transmission of this parasite among naïve indigenous breeds of bovines, particularly those owned by small-scale farmers, could lead to outbreaks similar to those which impacted the cattle industry in Australasia over the last decade [75,76].

*Babesia* was detected more in buffaloes (14.9%, 23/154) than in cattle (9.4%, 20/212), which might be explained by greater innate susceptibility of the former to piroplasms [63,77]. Interestingly, *Babesia* in buffaloes was found to a much greater extent (96%) in Sindh than in Punjab (4%), which might relate to genetic differences between Nili Ravi and Kundi breeds of buffalo in Punjab and Sindh provinces, respectively, and/or climatic distinctiveness between these two regions, with tick transmission being favoured in Sindh. The ‘host genetics’ proposal is supported somewhat by results from a Colombian study [78] showing a higher susceptibility of Murrah buffalo than Carabao and crossbred buffaloes to *Babesia* species. Another study [65] also indicated that the host genotype influences the impact of TBPs in bovines. Further work is required to explore and verify the differences in susceptibility to *Babesia* spp. between host species and breeds in endemic and non-endemic regions.

The relatively high level of genetic variation (up to 6.3% in 18S) within *B. bovis* accords with previous findings (also 18S) for species of *Babesia* [79], raising a pertinent question as to whether the distinct ASVs identified here within *B. bovis* represent population variants or cryptic species. This question warrants investigation using genomic sequencing and informatic tools, as inferences regarding species status based on variation in 18S alone may not be entirely unreliable [79,80,81,82].

This study records *T. lestoquardi*-like and *B. occultans* for the first time in bovines in Pakistan. Although found in buffalo, *T. lestoquardi* usually causes a malignant form of theileriosis in small ruminants in Pakistan and other parts of the world [83]. Since bovines are often reared together with sheep and goats and this pathogen shares the same tick vector as *T. annulata* (i.e., H. anatolicum), it is plausible that *T. lestoquardi* has jumped a small ruminant (via the tick) to a new host species (buffalo), similar to cases reported from Iran [84] and Sudan [85]. This is further substantiated by *T. lestoquardi* being the only piroplasm species of small ruminants which has possibly evolved from a cattle-infecting piroplasm species [86]. This hypothesis is supported by several features shared between both piroplasm species, including the high level of sequence homogeneity, antigenic cross-reactivity, overlapping distribution, and the same vector i.e., Hyalomma species [87,88]. Interestingly, *T. lestoquardi* does not appear to cause disease in bovines, but this warrants investigation. Also *B. occultans*—the other, new record in cattle for the Indian subcontinent—has been recognised as non-pathogenic [89], but was linked to a babesiosis outbreak in cattle in Italy [90]. This species has been recorded previously in H. anatolicum species, from cattle from the same district of Pakistan [91] as this new record in cattle, suggesting active, local transmission from tick to bovine, although this requires verification.

In conclusion, this study demonstrates the utility and benefits of using PCR-coupled NGS to directly explore piroplasm populations in host animals with mixed infections and to discover species or operational taxonomic units, undetectable using conventional techniques. Given its analytical sensitivity and specificity, this NGS approach should provide a powerful tool to explore temporal and spatial changes in piroplasm compositions in both livestock animals and associated tick vectors, and could also be used to assist in assessing the effectiveness of anti-piroplasm vaccines or the impact of host genotype (bovine species or breed) on susceptibility/resistance to piroplasm infections. More broadly, this tool could be adapted and applied to investigating piroplasm species of other host species and zoonotic representatives infecting humans.

## Figures and Tables

**Table 1:** Piroplasm species detected in bovines from seven districts of Punjab and Sindh, Pakistan.

Piroplasm Species	District(s)	Water Buffalo		Cattle	
		Male	Female	Male	Female
*Theileria annulata*	Okara	—	10/23	7/7	27/28
	Jhelum	3/7	7/18	—	19/22
	Bahawalpur	—	4/11	5/5	21/21
	Layyah	—	5/9	3/5	36/39
	Jhang	—	2/7	4/4	21/34
	Sukkur	5/9	9/19	8/8	18/19
	Thatta	4/13	10/28	4/5	9/11
*T. lestoquardi*-like	Thatta	1/13	—	—	—
*T. orientalis*	Okara	—	—	—	1/28
	Jhang	—	—	—	1/34
*Babesia bovis*	Okara	—	—	1/5	1/28
	Jhelum	—	—	—	4/22
	Bahawalpur	—	—	—	1/21
	Layyah	—	1/9	—	1/39
	Jhang	—	—	—	1/19
	Sukkur	1/9	4/19	1/5	2/11
	Thatta	3/13	5/28	—	—
*B. bigemina*	Okara	—	—	—	2/28
	Bahawalpur	—	—	2/5	—
	Layyah	—	—	—	—
	Sukkur	1/9	6/19	1/8	—
	Thatta	1/13	1/28	—	2/11
*B. occultans*	Layyah	—	—	—	1/39

Not detected (—).

## Supplementary Materials

The following are available online at https://www.mdpi.com/2076-2607/9/1/21/s1, Figure S1: Nucleotide alignment of the 18S rRNA amplicon sequence variants of *Babesia* species (*B. bovis*, BBo1-Bbo14; *B. occultans*, BOC1; *B. bigemina*, BBi1-BBi9). A dot indicates an identical nucleotide with respect to the top sequence. Figure S2: Nucleotide alignment of amplicon sequence variants (18S rRNA) of *Theileria* species (*T. orientalis*, TOR1-TOR4; *T. lestoquardi*-like, TLE1; *T. annulata*, TA1-TA146). A dot indicates an identical nucleotide with respect to the top sequence. Figure S3. Relative abundance of 18S sequences of *Babesia* species in individual bovine blood samples from distinct districts in Pakistan. Figure S4. Relative abundance of 18S sequences of *Theileria* species in individual bovine blood samples from distinct districts in Pakistan. Table S1: Pairwise comparison of 18S rRNA nucleotide amplicon sequence variant sequences belonging to *Theileria* (*T. orientalis*, TOR1-TOR4; *T. lestoquardi*-like, TLE1; *T. annulata*, TA1-TA146) species determined herein. Nucleotide similarity and percentage differences are given above and below the diagonal, respectively. Table S2: Pairwise comparison of 18S rRNA nucleotide amplicon sequence variant sequences belonging to *Babesia* species (*B. bovis*, BBo1-Bbo14; *B. occultans*, BOC1; *B. bigemina*, BBi1-BBi9) determined herein. Nucleotide similarity and percentage differences are given above and below the diagonal, respectively.
