# Supplementary material for: Targeted Next-Generation Sequencing and Informatics as an Effective Tool to Establish the Composition of Bovine Piroplasm Populations in Endemic Regions
Source: Microorganisms. 2020 Dec 23;9(1):21. doi: 10.3390/microorganisms9010021 (PMC7822421; doi:10.3390/microorganisms9010021)
Supplement: Supplementary file 1 [file microorganisms-09-00021-s001.zip › microorganisms-1024596 suppl/microorganisms-1024596 supplementary1.docx]

**Supplementary Data**

10 20 30 40 50 60 70 80 90 100

....|....|....|....|....|....|....|....|....|....|....|....|....|....|....|....|....|....|....|....|

MW165717_BBo1 CGGGGCTACTGCTCTGTAATTGGCATGGGGGCGACCTTCACCCTCGCCCGAGTACCCATTGGAGGGCAAGTCTGGTGCCAGCAGCCGCGGTAATTCCAGC

MW165718_BBo2 ....................................................................................................

MW165719_BBo3 ....................................................................................................

MW165720_BBo4 ....................................................................................................

MW165721_BBo5 ....................................................................................................

MW165722_BBo6 ....................................................................................................

MW165723_BBo7 ....................................................................................................

MW165724_BBo8 ....................................................................................................

MW165725_BBo9 ....................................................................................................

MW165726_BBo10 ....................................................................................................

MW165727_BBo11 ....................................................................................................

MW165728_BBo12 ....................................................................................................

MW165729_BBo13 ....................................................................................................

MW165730_BBo14 ....................................................................................................

MW165731_BOC1 .A.........TCT.........A...AT..T...T.AA......A..A.......A...........................................

MW165732_BBi1 .A.....TTC.TCT.........A...AT..T..TG.A..A....A..A.......A...........................................

MW165733_BBi2 .A.....TTC.TCT.........A...AT..T..TG.A..A....A..A.......A...........................................

MW165734_BBi3 .A.....TTC.TCT.........A...AT..T.GTG.A..A....A..A.......A...........................................

MW165735_BBi4 .A.....TTC.TCT.........A...AT..T..TG.A..A....A..AA......A...........................................

MW165736_BBi5 .A.....TTC.TCT.........A...AT..T..TG.A..A....A..A.......A...........................................

MW165737_BBi6 .A.....TTC.TCT.........A...AT..T..TG.A..A....A..A.......A...........................................

MW165738_BBi7 .A.....TTC.TCT.........A...AT..T..TG.A..A....A..A.......A...........................................

MW165739_BBi8 .A.....TTC.TCT.........A...AT..T..TG.A..A....A..A.......A...........................................

MW165740_BBi9 .A.....TTC.TCT.........A...AT..T..TG.A..A....A..A.......A...........................................

110 120 130 140 150 160 170 180 190 200

....|....|....|....|....|....|....|....|....|....|....|....|....|....|....|....|....|....|....|....|

MW165717_BBo1 TCCAATAGCGTATATTAAGCTTGTTGCAGTTAAAAAGCTCGTAGTTGTACTTTATGTCCCTCGC--GTGGTCC---------TT-CCTCG-CTAGGACGC

MW165718_BBo2 ..................A.............................................--.......---------..-.....-.........

MW165719_BBo3 ..................A.............................................--.......---------..-.....-.........

MW165720_BBo4 ..................A.............................................--.......---------..-.....-.........

MW165721_BBo5 ..................A..............G..............................--.......---------..-.....-.........

MW165722_BBo6 ..................A..............G..............................--.......---------..-.....-.........

MW165723_BBo7 ..................A...........................T......TC....TGT..--.......---------..T.....-.G-....A.

MW165724_BBo8 ..................A...........................T......TC....TGT..--.......---------..T.....-.G-....A.

MW165725_BBo9 ..................A...........................T......TC....TGT..--.......---------..T.....-.GC....A.

MW165726_BBo10 ..................A...........................T......TC....T-...TA...TGTT---------..CA..A.-GCGT...A.

MW165727_BBo11 ..................A...........................T......TC....T-...TA...TGTT---------..CA..A.-GCGT....G

MW165728_BBo12 ..................A...........................T......TC....T-...TA...TGTT---------..CA..A.-GCGT.....

MW165729_BBo13 ..................A............................A.TC.C.C.....G.C.TA...T-T.---------..CA..A.-GCG-.....

MW165730_BBo14 ..................A............................A.TC.C.C.....G.C.TA...T-T----------..CA..A.-GCGT...A.

MW165731_BOC1 ..................A............................A.T..C.-.CGTT....GT...CCT.--TTTTGGCCCAT....T..C.CTTTT

MW165732_BBi1 ..................A..............................T..C.-.C.T.G..TTTT.TCC.TGGTTTTGGG.CTTT...-..G.CTTT.

MW165733_BBi2 ..................A..............................T..C.-.C.T.G..TTTT.TCC.TGGTTTTGGG.CTTT...-..G.CTTTT

MW165734_BBi3 ..................A..............................T..C.-.C.T.G..TTTT.TCC.TGGTTTTGGG.CTTT...-..G.CTTTT

MW165735_BBi4 ..................A..............................T..C.-.C.T.G..TTTT.TCC.TGGTTTTGGG.CTTT...-..G.CTTTT

MW165736_BBi5 ..................A..............................T..C.-.C.T.G..TTTT.TCC.TGGTTTTGGG.CTTT...-..G.CTTTT

MW165737_BBi6 ..................A..............................T..C.-.C.T.G..TTTT.TCC.ACTTTTTGGG.CTTT...-..G.CTTT.

MW165738_BBi7 .................GA..............................T..C.-.C.T.G..TTTT.TCC.ACTTTTTGGG.CTTT...-..G.CTTT.

MW165739_BBi8 ..................A..............................T..CG-.C.T.G..TTTT.TCC.TCTTTTTGGG.CTTT...-..G.CTTTT

MW165740_BBi9 ..................A..............................T..C.-.C.T.G..TTTT.TCC.TCTTTTTGGG.CTTT...-..G.CTTTT

210 220 230 240 250 260 270 280 290 300

....|....|....|....|....|....|....|....|....|....|....|....|....|....|....|....|....|....|....|....|

MW165717_BBo1 CTCGTTACTTTGAGAAAATTAGAGTGTTTCAAGCAGGTTTCG--CCTGTATAATTGAGCATGGAATAACCTTGTATGACCCCTGTCGTACC--GTTGGTT

MW165718_BBo2 ..........................................--...............................................--.......

MW165719_BBo3 ..........................................--.....................................-.........--.......

MW165720_BBo4 ..........................................--...............................................--.......

MW165721_BBo5 ..........................................--..............T......................-.........--.......

MW165722_BBo6 ..........................................--.....................................-.........--.......

MW165723_BBo7 ........................................T.--.....................................-.........--.......

MW165724_BBo8 ........................................T.--.............................C.......-.........--.......

MW165725_BBo9 ........................................T.--.....................................-.........--.......

MW165726_BBo10 ........................................T.--.....................................-.........--.......

MW165727_BBo11 ........................................T.--.....................................-.........--.......

MW165728_BBo12 ........................................T.--.....................................-.........--.......

MW165729_BBo13 ........................................T.--.....................................-.........--.......

MW165730_BBo14 ........................................T.--.....................................-.........--.......

MW165731_BOC1 T.TA.................................C..TTGT.T..A...C...............TAGA...G....TTG..TC..TTTT.......

MW165732_BBi1 T.T-................................AC..TTGT.T..A...C..C............TAGA...G....TTG..TC..TTTT.......

MW165733_BBi2 T.T-................................AC..TTGT.T..A...C..C............TAGA...G....TTG..TC..TTTT.......

MW165734_BBi3 T.T-................................AC..TTGT.T..A...C..C............TAGA...G....TTG..TC..TTTT.......

MW165735_BBi4 T.T-................................AC..TTGT.T..A...C..C............TAGA...G....TTG..TC..TTTT.......

MW165736_BBi5 T.T-................................AC..TTGT.T..A...C..C............TAGA...G....TTG..TC..TTTT.......

MW165737_BBi6 T.T-................................AC..TTGT.T..A...C..C............TAGA...G....TTG..TC..TTTT.......

MW165738_BBi7 T.T-................................AC..TTGT.T..A...C..C............TAGA...G....TTG..TC..TTTT.......

MW165739_BBi8 T.T-................................AC..TTGT.T..A...C..C............TAGA...G....TTG..TC..TTTT.......

MW165740_BBi9 T.T-................................AC..TTGT.T..A...C..C............TAGA...G....TTG..TC..TTTT.......

310 320 330 340 350 360 370 380 390 400

....|....|....|....|....|....|....|....|....|....|....|....|....|....|....|....|....|....|....|....|

MW165717_BBo1 --GA-CTTTGGGTAATGGTTAATAGGAACGGTTGGGGGCATTCGTACTCGACTGTCAGAGGTGAAATTCTTAGATTTGTCGATGACGCACGACTGCGAAA

MW165718_BBo2 --..-...............................................................................................

MW165719_BBo3 --..-...............................................................................................

MW165720_BBo4 --..-...............................................................................................

MW165721_BBo5 --..-...............................................................................................

MW165722_BBo6 --..-...............................................................................................

MW165723_BBo7 --.--.C.............................................................................................

MW165724_BBo8 --.--.C.............................................................................................

MW165725_BBo9 --.--.C.............................................................................................

MW165726_BBo10 --.--.C.............................................................................................

MW165727_BBo11 --.--.C.............................................................................................

MW165728_BBo12 --.--.C.............................................................................................

MW165729_BBo13 --.--.C.............................................................................................

MW165730_BBo14 --.--.C.............................................................................................

MW165731_BOC1 TT.TTACC.T....................................T.TA.............................TA.A....A..T.........

MW165732_BBi1 TT..-GCC.T....................................T.TA.............................TA.A....A..C.........

MW165733_BBi2 TC..-GCC.T....................................T.TA.............................TA.A....A..C.........

MW165734_BBi3 TT..-GCC.T....................................T.TA.............................TA.A....A..C.........

MW165735_BBi4 TT..-GCC.T....................................T.TA.............................TA.A....A..C.........

MW165736_BBi5 TT..-GCC.T....................................T.TA.............................TA.A....A..C.........

MW165737_BBi6 TT..-GCC.T....................................T.TA.............................TA.A....A..C.........

MW165738_BBi7 TT..-GCC.T....................................T.TA.............................TA.A....A..C.........

MW165739_BBi8 TT..-GCC.T....................................T.TA.............................TA.A....A..C.........

MW165740_BBi9 TT..-GCC.T....................................T.TA.............................TA.A....A..C.........

410 420 430

....|....|....|....|....|....|....

MW165717_BBo1 GCATTTGCCAAGGACGCTTCCATTAATCAAGAAC

MW165718_BBo2 ....C.............................

MW165719_BBo3 ..................................

MW165720_BBo4 ..................................

MW165721_BBo5 ..................................

MW165722_BBo6 ..................................

MW165723_BBo7 ..................................

MW165724_BBo8 ..................................

MW165725_BBo9 ..................................

MW165726_BBo10 ..................................

MW165727_BBo11 ..................................

MW165728_BBo12 ..................................

MW165729_BBo13 ..................................

MW165730_BBo14 ..................................

MW165731_BOC1 ................T.................

MW165732_BBi1 ................T..T..............

MW165733_BBi2 ................T..T..............

MW165734_BBi3 ................T..T..............

MW165735_BBi4 ................T..T..............

MW165736_BBi5 ................T..T..............

MW165737_BBi6 ................T..T..............

MW165738_BBi7 ................T..T..............

MW165739_BBi8 ................T..T..............

MW165740_BBi9 ................T..T..............

**Figure S1.** Nucleotide alignment of the 18S rRNA amplicon sequence variants of *Babesia* species (*B. bovis*, BBo1-Bbo14; *B. occultans,* BOC1; *B. bigemina,* BBi1-BBi9). A dot indicates an identical nucleotide with respect to the top sequence.

10 20 30 40 50 60 70 80 90 100

....|....|....|....|....|....|....|....|....|....|....|....|....|....|....|....|....|....|....|....|

MW165707_TOR1 CGGGGCTTAATGTCTTGTAATTGGAATGATGGGAATTTAAACCTCTTCCAGAGTATCAATTGGAGGGCAAGTCTGGTGCCAGCAGCCGCGGTAATTCCAG

MW165708_TOR2 ....................................................................................................

MW165709_TOR3 ....................................................................................................

MW165710_TOR4 ....................................................................................................

MW165716_TLE1 ..........A.........................................................................................

MW165561_TA1 ..........A.........................................................................................

MW165562_TA2 ..........A..................................C......................................................

MW165563_TA3 ..........A.........................................................................................

MW165564_TA4 ..........A.........................................................................................

MW165565_TA5 ..........A.........................................................................................

MW165566_TA6 ..........A.........................................................................................

MW165567_TA7 ..........A.........................................................................................

MW165568_TA8 ..........A.........................................................................................

MW165569_TA9 ..........A.........................................................................................

MW165570_TA10 ..........A.........................................................................................

MW165571_TA11 .T........A.........................................................................................

MW165572_TA12 ..........A.........................................................................................

MW165573_TA13 ..........A.........................................................................................

MW165574_TA14 ..........A.........................................................................................

MW165575_TA15 ..........A...........AT............................................................................

MW165576_TA16 ..........A................................CT.......................................................

MW165577_TA17 ..........A.............................T..G........................................................

MW165578_TA18 ..........A.........................................................................................

MW165579_TA19 ..........A.........................................................................................

MW165580_TA20 ..........A.........................................................................................

MW165581_TA21 ..........A.........................................................................................

MW165582_TA22 ..........A.........................................................................................

MW165583_TA23 ..........A.........................................................................................

MW165584_TA24 ..........A.........................................................................................

MW165585_TA25 ..........A.........................................................................................

MW165586_TA26 ..........A.........................................................................................

MW165587_TA27 ..........A.........................................................................................

MW165588_TA28 ..........A.........................................................................................

MW165589_TA29 ..........A..............................T..........................................................

MW165590_TA30 ..........A..............................TT.........................................................

MW165591_TA31 ..........A...............................A.........................................................

MW165592_TA32 ..........A.........................................................................................

MW165593_TA33 ..........A.........................................................................................

MW165594_TA34 ..........A.........................................................................................

MW165595_TA35 ..........A.........................................................................................

MW165596_TA36 .....A....A.........................................................................................

MW165597_TA37 ..........A.........................................................................................

MW165598_TA38 ..........A.........................................................................................

MW165599_TA39 ..........A.........................................................................................

MW165600_TA40 ..........A.........................................................................................

MW165601_TA41 ..........G.........................................................................................

MW165602_TA42 ....................................................................................................

MW165603_TA43 ....................................................................................................

MW165604_TA44 ..........A.............................................T...........................................

MW165605_TA45 ..........A.........................................................................................

MW165606_TA46 ..........A.........................................................................................

MW165607_TA47 ..........A.........................................................................................

MW165608_TA48 ..........A.........................................................................................

MW165609_TA49 ..........A.........................................................................................

MW165610_TA50 ........T.A.........................................................................................

MW165611_TA51 ..........A.........................A...............................................................

MW165612_TA52 ........G.A.........................................................................................

MW165613_TA53 ..........A.........................................................................................

MW165614_TA54 ..........A.........................C...............................................................

MW165615_TA55 ..........A.........................................................................................

MW165616_TA56 ..........A.........................................................................................

MW165617_TA57 ..........A.........................................................................................

MW165618_TA58 ..........A.........................................................................................

MW165619_TA59 ..........A.........................................................................................

MW165620_TA60 ..........A.........................................................................................

MW165621_TA61 ..........A.........................................................................................

MW165622_TA62 ..........A.........................................................................................

MW165623_TA63 ..........A.........................................................................................

MW165624_TA64 ..........A.........................................................................................

MW165625_TA65 ..........A.........................................................................................

MW165626_TA66 ..........A.........................................................................................

MW165627_TA67 ..........A.........................................................................................

MW165628_TA68 ..........A.........................................................................................

MW165629_TA69 ..........A.........................................................................................

MW165630_TA70 ..........A.........................................................................................

MW165631_TA71 ..........A.........................................................................................

MW165632_TA72 ..........A.........................................................................................

MW165633_TA73 ..........A.........................................................................................

MW165634_TA74 ..........A.........................................................................................

MW165635_TA75 ..........A.........................................................................................

MW165636_TA76 ..........A.........................................................................................

MW165637_TA77 .A........A.........................................................................................

MW165638_TA78 ..........A.........................................................................................

MW165639_TA79 ..........A.........................................................................................

MW165640_TA80 ..........A.........................................................................................

MW165641_TA81 ..........A.........................................................................................

MW165642_TA82 ..........A.........................................................................................

MW165643_TA83 ..........A.........................................................................................

MW165644_TA84 ..........A.........................................................................................

MW165645_TA85 ..........A.........................................................................................

MW165646_TA86 ..........A.........................................................................................

MW165647_TA87 ..........A.........................................................................................

MW165648_TA88 ..........A.........................................................................................

MW165649_TA89 ..........A.........................................................................................

MW165650_TA90 ..........A.........................................................................................

MW165651_TA91 ..........A.........................................................................................

MW165652_TA92 ..........A.........................................................................................

MW165653_TA93 ..........A.........................................................................................

MW165654_TA94 ..........A.........................................................................................

MW165655_TA95 ..........A.........................................................................................

MW165656_TA96 ..........A.........................................................................................

MW165657_TA97 ..........A.........................................................................................

MW165658_TA98 ..........A.........................................................................................

MW165659_TA99 ..........A.........................................................................................

MW165660_TA100 ..........A.........................................................................................

MW165661_TA101 ..........A.........................................................................................

MW165662_TA102 ..........A.........................................................................................

MW165663_TA103 ..........A.........................................................................................

MW165664_TA104 ..........A.........................................................................................

MW165665_TA105 ..........A.........................................................................................

MW165666_TA106 ..........A.................T.......................................................................

MW165667_TA107 ..........A..............G..........................................................................

MW165668_TA108 ..........A.........................................................................................

MW165669_TA109 ..........A.........................................................................................

MW165670_TA110 ..........A.........................................................................................

MW165671_TA111 ..........A...........T.............................................................................

MW165672_TA112 ..........A.................................A.......................................................

MW165673_TA113 ..........A.........................................................................................

MW165674_TA114 ..........A.........................................................................................

MW165675_TA115 ..........A...............................G.........................................................

MW165676_TA116 .......A..A.........................................................................................

MW165677_TA117 ..........A.........................................................................................

MW165678_TA118 ..........A...........................T.............................................................

MW165679_TA119 ..........A.........................................................................................

MW165680_TA120 ..........A.........................................................................................

MW165681_TA121 ..........A.......................T.................................................................

MW165682_TA122 ..........A.........................................................................................

MW165683_TA123 ..........A.........................................................................................

MW165684_TA124 ..........A.........................................................................................

MW165685_TA125 ..........A.........................................................................................

MW165686_TA126 ..........A.........................................................................................

MW165687_TA127 ..........A.........................................................................................

MW165688_TA128 ..........A.........................................................................................

MW165689_TA129 ......G...A.........................................................................................

MW165690_TA130 ..........A.........................................................................................

MW165691_TA131 ..........A.........................................................................................

MW165692_TA132 ..........A.........................................................................................

MW165693_TA133 ..........A.........................................................................................

MW165694_TA134 ..........A.........................................................................................

MW165695_TA135 ..........A.........................................................................................

MW165696_TA136 ..........A.........................................................................................

MW165697_TA137 ..........A.........................................................................................

MW165698_TA138 ..........A.........................................................................................

MW165699_TA139 ..........A.........................................................................................

MW165700_TA140 ..........A..................................................A......................................

MW165701_TA141 ..........A.........................................................................................

MW165702_TA142 ..........A........................C................................................................

MW165703_TA143 ..........A...A.....................................................................................

MW165704_TA144 ..........A.........................................................................................

MW165705_TA145 ..........A.........................................................................................

MW165706_TA146 ..........A.........................................................................................

110 120 130 140 150 160 170 180 190 200

....|....|....|....|....|....|....|....|....|....|....|....|....|....|....|....|....|....|....|....|

MW165707_TOR1 CTCCAATAGCGTATATTAAAATTGTTGCAGTTAAAAAGCTCGTAGTTGAATTTCTGCTGCATTACATATCTCTTGTTTGAGTTTGTTTTTGTGGCTTATT

MW165708_TOR2 ....................T..............................................T...................A............

MW165709_TOR3 ....................T..............................................T...........G.......A............

MW165710_TOR4 ....................T..............................................T...................A............

MW165716_TLE1 ...............................................................G.T.G.G..CCTC.G.G..C...GCA........T..

MW165561_TA1 ...............................................................G.T.G.G..CCT....G..C...GCA........T..

MW165562_TA2 ...............................................................G.T.G.G..CCT..G.G..C...GCA........T..

MW165563_TA3 ...............................................................G.T.G.G..CCT..G.G..C...GCA........T..

MW165564_TA4 ...............................................................G.T.G.G..CCTC.G.G..C...GCA........T..

MW165565_TA5 ...............................................................G.T.G.G..CCTC...G..C...GCA........T..

MW165566_TA6 ...............................................................G.T.G.G..CCTC...G..C.C.GCA........T..

MW165567_TA7 ...............................................................G.T.G.G..CCTC...G..C.T.GCA........T..

MW165568_TA8 ...............................................................G.T.G.G..CCTC...G..C.A.GCA........T..

MW165569_TA9 ...............................................................G.T.G.G..CCTC.G.G..C.A.GCA........T..

MW165570_TA10 ...............................................................G.T.G.G..CCTC.G.G..C.T.GCA........T..

MW165571_TA11 ...............................................................G.T.G.G..CCTC...G..C...GCA........T..

MW165572_TA12 ...............................................................G.T.G.G..CCTC.C.G..C...GCA........T..

MW165573_TA13 ...............................................................G.T.G.G..CCTC...G..C...GCA........T..

MW165574_TA14 ...............................................................G.T.G.G..CCTC...G..C...GCA........T..

MW165575_TA15 ...............................................................G.T.G.G..CCTC.G.G..C...GCA........T..

MW165576_TA16 ...............................................................G.T.G.G..CCTC.G.G..C...GCA........T..

MW165577_TA17 ...............................................................G.T.G.G..CCTC.G.G..C...GCA........T..

MW165578_TA18 ...............................................................G.T.G.G..CCTC.G.G..C...GCA........T..

MW165579_TA19 ...............................................................G.T.G.G..CCTC.G.G..C...GCA........T..

MW165580_TA20 ...............................................................G.T.G.G..CCTC.G.G..C...GCA........T..

MW165581_TA21 ...............................................................G.T.G.G..CCTC.G.G..C...GCA........TC.

MW165582_TA22 ...............................................................G.T.G.G..CCTC.G.G..C...GCA........T..

MW165583_TA23 ...............................................................G.T.G.G..CCTC.G.G..C...GCA........T..

MW165584_TA24 ...............................................................G.T.G.G..CCTC.G.G..C...GCA........T..

MW165585_TA25 ...............................................................G.T.G.G..CCTC.G.G..C...GCA........T..

MW165586_TA26 ...............................................................G.T.G.G..CCTC.G.G..C...GCA........T..

MW165587_TA27 ...............................................................G.T.G.G..CCTC.G.G..C...GCA........T..

MW165588_TA28 ...............................................................G.T.G.G..CCTC.G.G..C...GCA........T..

MW165589_TA29 ...............................................................G.T.G.G..CCTC.G.G..C...GCA........T..

MW165590_TA30 ...............................................................G.T.G.G..CCTC.G.G..C...GCA........T..

MW165591_TA31 ...............................................................G.T.G.G..CCTC.G.G..C...GCA.A......T..

MW165592_TA32 ...............................................................G.T.G.G..CCTC.G.G..C...GCA.A......T..

MW165593_TA33 ...............................................................G.T.G.G..CCTC.G.G..C...GCA.TC.....T..

MW165594_TA34 ...............................................................G.T.G.G..CCTC.G.G..C...GCA..C.....T..

MW165595_TA35 ...............................................................G.T.G.G..CCTC.G.G......GCA........T..

MW165596_TA36 ...............................................................G.T.G.G..CCTC.G.G..A...GCA........T..

MW165597_TA37 ...............................................................G.T.G.G..CCTC.G.G..A...GCA........T..

MW165598_TA38 ...............................................................GA..G.G..CCTC.G.G..C...GCA........T..

MW165599_TA39 ...............................................................G.C.G.G..CCTC.G.G..C...GCA........T..

MW165600_TA40 ...............................................................G...G.G..CCTC.G.G..C...GCA........T..

MW165601_TA41 ...............................................................G.T.G.G..CCTC.G.G..C...GCA........T..

MW165602_TA42 ...............................................................C.T.G.G..CCTC.G.G..C...GCA........T..

MW165603_TA43 ...............................................................G.T.G.G..CCTC.G.G..C...GCA........T..

MW165604_TA44 ...............................................................G.T.G.G..CCTC.G.G..C...GCA.....T..T..

MW165605_TA45 ...............................................................G.T.G.G..CCTC.G.G..C...GCA.....G..T..

MW165606_TA46 ...............................................................G.T.G.G..CCTC.G.G..C...GCA.....T..T..

MW165607_TA47 ...............................................................G.T.G.G..CCTC.G.G..C...GCA........T..

MW165608_TA48 ...............................................................G.T.G.G..CCTC.G.G..C...GCA........T..

MW165609_TA49 ...............................................................G.T.G.G..CCTC.G.G..C...GCA........T..

MW165610_TA50 ...............................................................G.T.G.G..CCTC.G.G..C...GCA........T..

MW165611_TA51 ...............................................................G.T.G.G..CCTC.G.G..C...GCA........T..

MW165612_TA52 ...............................................................G.T.G.G..CCTC.G.G..C...GCA........T..

MW165613_TA53 ...............................................................G.T.G.G..CCTC.G.G..C...GCA........T..

MW165614_TA54 ...............................................................G.T.G.G..CCTC.G.G..C...GCA........T..

MW165615_TA55 ...............................................................G.T.G.G..CCTC.G.G..C...GCA........T..

MW165616_TA56 ...............................................................G.T.G.G..CCTC.G.G..C...GCA........T..

MW165617_TA57 ...............................................................G.T.G.G..CCTC.G.G..C...GCA........T..

MW165618_TA58 ...............................................................G.T.G.G..CCTC.G.G..C...GCA........T..

MW165619_TA59 ...............................................................G.T.G.G..CCTC.G.G..C...GCA........T..

MW165620_TA60 ...............................................................G.T.G.G..CCTC.G.G..C...GCA........T..

MW165621_TA61 ...............................................................G.T.G.G..CCTC.G.G..C...G.A........T..

MW165622_TA62 ...............................................................G.T.G.G..CCTC.G.G..C...GCA........T..

MW165623_TA63 ...............................................................G.T.G.G..CCTC.G.G..C...GCA........T..

MW165624_TA64 ...............................................................G.T.G.G..CCTC.G.G..C...GCA........T..

MW165625_TA65 ...............................................................G.T.G.G..CCTC.G.G..C...GAA........T..

MW165626_TA66 ...............................................................GTT.G.G..CCTC.G.G..C...GCA........T..

MW165627_TA67 ...............................................................GAT.G.G..CCTC.G.G..C...GCA........T..

MW165628_TA68 ...............................................................G.TAG.G..CCTC.G.G..C...GCA........T..

MW165629_TA69 ...............................................................T.T.G.G..CCTC.G.G..C...GCA........T..

MW165630_TA70 ...............................................................G.TCG.G..CCTC.G.G..C...GCA........T..

MW165631_TA71 ...............................................................C.T.G.G..CCTC.G.G..C...GCA........T..

MW165632_TA72 ...............................................................G.T.G.G..CCTC.G.G..C...GCA........T..

MW165633_TA73 ...............................................................G.T.G.G..CCTC.G.G..C...GCA........T..

MW165634_TA74 ...............................................................G.T.G.G..CCTC.GTG..C...GCA........T..

MW165635_TA75 ...............................................................G.T.G.G..CCTC.GCG..C...GCA........T..

MW165636_TA76 ...............................................................G.T.G.G..CCTC.GAG..C...GCA........T..

MW165637_TA77 ...............................................................G.T.G.G..CCTC.G.G..C...GCA........T..

MW165638_TA78 ...............................................................G.T.G.G..CCTC.G.G..C...GCA........T..

MW165639_TA79 ...............................................................G.T.G.G..CCTC.G.G..C...GCA........T..

MW165640_TA80 ...............................................................G.T.G.G..CCTC.G.G..C...GCA........T..

MW165641_TA81 ...............................................................G.T.G.G..CCTC.G.G..C...GCA........T..

MW165642_TA82 ...............................................................G.T.G.G...CTC.G.G..C...GCA........T..

MW165643_TA83 ...............................................................G.T.G.G..CCTC.G.G..C...GCG........T..

MW165644_TA84 ...............................................................G.T.T.G..CCTC.G.G..C...GCA........T..

MW165645_TA85 ...............................................................G.T.G.G..CCTC.G.G..C...GCA........T..

MW165646_TA86 ...................................G...........................G.T.G.G..CCTC.G.G..C...GCA........T..

MW165647_TA87 ...............................................................G.T.G.G..CCTC.G.G..C...GCA........T..

MW165648_TA88 ...............................................................G.T.G.G..CCTC.G.G..C...GCA........T..

MW165649_TA89 .........................................................C.....G.T.G.G..CCTC.G.G..C...GCA........T..

MW165650_TA90 ....................T..........................................G.T.G.G..CCTC.G.G..C...GCA........T..

MW165651_TA91 ...............................................................G.T.G.G..C.TC.G.G..C...GCA........T..

MW165652_TA92 .............................................................A.G.T.G.G..CCTC.G.G..C...GCA........T..

MW165653_TA93 ...............................................A...............G.T.G.G..CCTC.G.G..C...GCA........T..

MW165654_TA94 ...............................................................G.T.G.G..CCTC.G.G..C...GCA........T..

MW165655_TA95 ...............................................................G.T.G.G..CCTC.G.G..C...GCA........T..

MW165656_TA96 ...............................................................G.T.G.G..CCTC.G.G..C...GCA........T..

MW165657_TA97 ...............................................................G.T.G.G..CCTC.G.G..C...GCA........T..

MW165658_TA98 ...............................................................G.T.G.G..CCCC.G.G..C...GCA........T..

MW165659_TA99 ...............................................................G.T.G.G..CCTC.G.G..C...GCA........T..

MW165660_TA100 ....................................G..........................G.T.G.G..CCTC.G.G..C...GCA........T..

MW165661_TA101 ..........................................................C....G.T.G.G..CCTC.G.G..C...GCA........T..

MW165662_TA102 ...............................................................G.T.G.G..CCTC.G.G..C...GCA........T..

MW165663_TA103 ...............................................................G.T.G.G..CCTC.G.G..C...GCA........T..

MW165664_TA104 ...............................................................G.T.G.G..CCTC.G.G..C...GCA........T..

MW165665_TA105 ...............................................................G.T.G.G..CCTC.G.G..C...GCA........T..

MW165666_TA106 ...............................................................G.T.G.G..CCTC.G.G..C...GCA........T..

MW165667_TA107 ...............................................................G.T.G.G..CCTC.G.G..C...GCA........T..

MW165668_TA108 ...............................................................G.T.G.G..CCTC.G.G..C...GCA...T....T..

MW165669_TA109 ...............................................................G.T.G.G..CCTC.G.G..C...GCA........T..

MW165670_TA110 ...............................................................G.T.G.G..CCTC.G.G..C...GCA........T..

MW165671_TA111 ...............................................................G.T.G.G..CCTC.G.G..C...GCA........T..

MW165672_TA112 ...............................................................G.T.G.G..CCTC.G.G..C...GCA........T..

MW165673_TA113 ...............................................................G.T.G.G..CCTC.G.G..C...GCA........T..

MW165674_TA114 ...............................................................G.T.G.G..CCTC.G.G..C...GCA........T..

MW165675_TA115 ...............................................................G.T.G.G..CCTC.G.G..C...GCA........T..

MW165676_TA116 ...............................................................G.T.G.G..CCTC.G.G..C...GCA........T..

MW165677_TA117 ...............................................................G.T.G.G..CCTC.G.G..C...GCA........T..

MW165678_TA118 ...............................................................G.T.G.G..CCTC.G.G..C...GCA........T..

MW165679_TA119 ...............................................................G.T.G.G..CCTC.G.G..C...GCA........T..

MW165680_TA120 ...............................................................G.T.G.G..CCTC.G.G..C...GCA........T..

MW165681_TA121 ...............................................................G.T.G.G..CCTC.G.G..C...GCA........T..

MW165682_TA122 ...............................................................G.T.G.G..CCTC.G.G..C...GCA........T..

MW165683_TA123 ...........................T...................................G.T.G.G..CCTC.G.G..C...GCA........T..

MW165684_TA124 ....G..........................................................G.T.G.G..CCTC.G.G..C...GCA........T..

MW165685_TA125 ...............................................................G.T.G.G..CCTC.G.G..C...GCA........T..

MW165686_TA126 ...............................................................G.T.G.GC.CCTC.G.G..C...GCA........T..

MW165687_TA127 ...............................................................G.T.G.T..CCTC.G.G..C...GCA........T..

MW165688_TA128 ...............................................................G.T.G.G..CCTC.G.G..C...GCA........T..

MW165689_TA129 ...............................................................G.T.G.G..CCTC.G.G..C...GCA........T..

MW165690_TA130 ...............................................................G.T.G.G..CCTC.G.G..C...GCA........T..

MW165691_TA131 ...............................................................G.T.G.G..CCTC.G.G..C...GCA........T..

MW165692_TA132 ...............................................................G.T.G.G..CCTC.G.G..C...GCA........T..

MW165693_TA133 ...............................................................G.T.G.G..CCTC.G.G..C...GCA........T..

MW165694_TA134 ...............................................................G.T.G.G..CCTC.G.G..C..CGCA........T..

MW165695_TA135 ...............................................................G.T.G.G..CCTC.G.G..C...GCA........T..

MW165696_TA136 ...............................................................G.T.G.G..CCTC.G.G..C...GCA........T..

MW165697_TA137 ...............................................................G.T.G.G..CCTC.G.G..C...GCA........T..

MW165698_TA138 ................................G..............................G.T.G.G..CCTC.G.G..C...GCA........T..

MW165699_TA139 ...............................................................G.T.G.G..CCTC.G.G..C...GCA........T..

MW165700_TA140 ...............................................................G.T.G.G..CCTC.G.G..C...GCA........T..

MW165701_TA141 ...............................................................G.T.G.G..CCTC.G.G..C...GCA........T..

MW165702_TA142 ...............................................................G.T.G.G..CCTC.G.G..C...GCA........T..

MW165703_TA143 ...............................................................G.T.G.G..CCTC.G.G..C...GCA........T..

MW165704_TA144 ...............................................................G.T.G.G..CCTC.G.G..C...GCA........T..

MW165705_TA145 ...............................................................G.T.G.G..CCTC.G.G..C...GCA........T..

MW165706_TA146 ...............................................................G.T.G.G..CCTC.G.G..C...GCA........T..

210 220 230 240 250 260 270 280 290 300

....|....|....|....|....|....|....|....|....|....|....|....|....|....|....|....|....|....|....|....|

MW165707_TOR1 TCGGTTTGATTTTT-TCTTTCCGGATGATTACTTTGAGAAAATTAGAGTGCTCAAAGCAGGCTTTCGCCTTGAATAGTTTAGCATGGAATAATAAAGTAG

MW165708_TOR2 ....A.........A..................................................T..........C.................G.....

MW165709_TOR3 ....A.........A..................................................T............................G.....

MW165710_TOR4 ....A.........A..................................................T............................G.....

MW165716_TLE1 ....ACG..G...C-.T.G..T.A...T........................TC...........T..........C................GG.....

MW165561_TA1 ....ACG..G...C-.T.G..T.A...T........................................................................

MW165562_TA2 ....ACG..G...C-.T.G..T.A...T........................................................................

MW165563_TA3 ....ACG..G...C-.T.G..T.A...T........................................................................

MW165564_TA4 ....ACG.GG...C-.T.G..T.A...T........................................................................

MW165565_TA5 ....ACG.GG...C-.T.G..T.A...T........................................................................

MW165566_TA6 ....ACG..G...C-.T.G..T.A...T........................................................................

MW165567_TA7 ....ACG..G...C-.T.G..T.A...T........................................................................

MW165568_TA8 ....ACG..G...C-.T.G..T.A...T........................................................................

MW165569_TA9 ....ACG..G...C-.T.G..T.A...T........................................................................

MW165570_TA10 ....ACG..G...C-.T.G..T.A...T........................................................................

MW165571_TA11 ....ACG..G...C-.T.G..T.A...T........................................................................

MW165572_TA12 ....ACG..G...C-.T.G..T.A...T........................................................................

MW165573_TA13 ....ACG..G...C-.T.G..T.A...T........................................................................

MW165574_TA14 ....ACG..G...C-.T.G..T.A...C........................................................................

MW165575_TA15 ....ACG..G...C-.T.G..T.A...T........................................................................

MW165576_TA16 ....ACG..G...C-.T.G..T.A...T........................................................................

MW165577_TA17 ....ACG..G...C-.T.G..T.A...T........................................................................

MW165578_TA18 ....AC...G...C-.T.G..T.A...T........................................................................

MW165579_TA19 ....ACG..G...C-.T.G....A...T........................................................................

MW165580_TA20 ....AC...G...C-.T.G....A...T........................................................................

MW165581_TA21 ....ACA..G...C-.T.G..T.A...T........................................................................

MW165582_TA22 ....ACA..G...C-.T.G..T.A...T........................................................................

MW165583_TA23 ....ACAA.G...C-.T.G..T.A...T........................................................................

MW165584_TA24 ....ACGC.G...C-.T.G..T.A...T........................................................................

MW165585_TA25 ....ACGA.G...C-.T.G..T.A...T........................................................................

MW165586_TA26 ..AAACG..G...C-.T.G..T.A...T........................................................................

MW165587_TA27 ..A.ACG..G...C-.T.G..T.A...T........................................................................

MW165588_TA28 ..A.ACG..G...C-.T.G..T.A...T........................................................................

MW165589_TA29 ....ACG..G...C-.T.G..T.A...T........................................................................

MW165590_TA30 ....ACG..G...C-.T.G..T.A...T........................................................................

MW165591_TA31 ....ACG..G...C-.T.G..T.A...T........................................................................

MW165592_TA32 ....ACG..G...C-.T.G..T.A...T........................................................................

MW165593_TA33 ....ACG..G...C-.T.G..T.A...T........................................................................

MW165594_TA34 ....ACG..G...C-.T.G..T.A...T........................................................................

MW165595_TA35 ....ACG..G...C-.T.G..T.A...T........................................................................

MW165596_TA36 ....ACG..G...C-.T.G..T.A...T........................................................................

MW165597_TA37 ....ACG..G...C-.T.G..T.A...T........................................................................

MW165598_TA38 ....ACG..G...C-.T.G..T.A...T........................................................................

MW165599_TA39 ....ACG..G...C-.T.G..T.A...T........................................................................

MW165600_TA40 ....ACG..G...C-.T.G..T.A...T........................................................................

MW165601_TA41 ....ACG..G...C-.T.G..T.A...T........................................................................

MW165602_TA42 ....ACG..G...C-.T.G..T.A...T........................................................................

MW165603_TA43 ....ACG..G...C-.T.G..T.A...T........................................................................

MW165604_TA44 ....ACG..G...C-.T.G..T.A...T........................................................................

MW165605_TA45 ....ACG..G...C-.T.G..T.A...T........................................................................

MW165606_TA46 ....ACG..G...C-.T.G..T.A...T........................................................................

MW165607_TA47 ....ACG..G...C-.T.G..T.A..TT........................................................................

MW165608_TA48 ....ACG..G...C-.T.G..T.A..CT........................................................................

MW165609_TA49 ....ACG..G...C-.T.G..T.A..AT........................................................................

MW165610_TA50 ....ACG..G...C-.T.G..T.A...T........................................................................

MW165611_TA51 ....ACG..G...C-.T.G..T.A...T........................................................................

MW165612_TA52 ....ACG..G...C-.T.G..T.A...T........................................................................

MW165613_TA53 ....ACG..G...C-.T.G..T.A...T....................................C...................................

MW165614_TA54 ....ACG..G...C-.T.G..T.A...T........................................................................

MW165615_TA55 ....ACG..G...C-.T.G..T.A...T....................................A...................................

MW165616_TA56 ....ACG..G...C-.T.G..T.A...T......................................A.................................

MW165617_TA57 ....ACG..G...C-.T.G..T.A...T......................................T.................................

MW165618_TA58 ....ACG..G...C-.T.G..T.A...T........................................................................

MW165619_TA59 ....ACG..G...C-.T.G..T.A...T........................................................................

MW165620_TA60 ....ACG..G...C-.T.G..T.AC..T........................................................................

MW165621_TA61 ....ACG..G...C-.T.G..T.A...T........................................................................

MW165622_TA62 ....ACG..G...C-.T.G..T.AT..T........................................................................

MW165623_TA63 ....ACG..G...C-.T.G..T.A...T........................................................................

MW165624_TA64 ....ACG..G...C-.T.G..T.A...T........................................................................

MW165625_TA65 ....ACG..G...C-.T.G..T.A...T........................................................................

MW165626_TA66 ....ACG..G...C-.T.G..T.A...T........................................................................

MW165627_TA67 ....ACG..G...C-.T.G..T.A...T........................................................................

MW165628_TA68 ....ACG..G...C-.T.G..T.A...T........................................................................

MW165629_TA69 ....ACG..G...C-.T.G..T.A...T........................................................................

MW165630_TA70 ....ACG..G...C-.T.G..T.A...T........................................................................

MW165631_TA71 ....ACG..G...C-.T.G..T.A...T........................................................................

MW165632_TA72 ....ACG......C-.T.G..T.A...T........................................................................

MW165633_TA73 ....ACG..A...C-.T.G..T.A...T........................................................................

MW165634_TA74 ....ACG..G...C-.T.G..T.A...T........................................................................

MW165635_TA75 ....ACG..G...C-.T.G..T.A...T........................................................................

MW165636_TA76 ....ACG..G...C-.T.G..T.A...T........................................................................

MW165637_TA77 ....ACG..G...C-.T.G..T.A...T........................................................................

MW165638_TA78 ....ACG..G...C-.T.G..T.A...T...............................T........................................

MW165639_TA79 ....ACG..G...C-.T.G..T.A...T.................................................................G......

MW165640_TA80 ....ACG..G...C-.T.G..T.A...T........................................................................

MW165641_TA81 ....ACG..G...C-.T.G..T.A...T........................................................................

MW165642_TA82 ....ACG..G...C-.T.G..T.A...T........................................................................

MW165643_TA83 ....ACG..G...C-.T.G..T.A...T........................................................................

MW165644_TA84 ....ACG..G...C-.T.G..T.A...T........................................................................

MW165645_TA85 ....ACG..G...C-.T.G..T.A...T........................................................................

MW165646_TA86 ....ACG..G...C-.T.G..T.A...T........................................................................

MW165647_TA87 ....ACG..G...C-.T.G..T.A...T........................................................................

MW165648_TA88 ....ACG..G...C-.T.G..T.A...T........................................................................

MW165649_TA89 ....ACG..G...C-.T.G..T.A...T........................................................................

MW165650_TA90 ....ACG..G...C-.T.G..T.A...T........................................................................

MW165651_TA91 ....ACG..G...C-.T.G..T.A...T........................................................................

MW165652_TA92 ....ACG..G...C-.T.G..T.A...T........................................................................

MW165653_TA93 ....ACG..G...C-.T.G..T.A...T........................................................................

MW165654_TA94 ....ACG..G...C-...G..T.A...T........................................................................

MW165655_TA95 ....ACG..G...C-.T.G..T.A...T...........................................T............................

MW165656_TA96 ....ACG..G...C-.T.G..T.A...T........................T...............................................

MW165657_TA97 ....ACG..G...C-.T.G..T.A...T........................................................................

MW165658_TA98 ....ACG..G...C-.T.G..T.A...T........................................................................

MW165659_TA99 ....ACG..G...C-.T.G..T.A...T.......................................................G................

MW165660_TA100 ....ACG..G...C-.T.G..T.A...T........................................................................

MW165661_TA101 ....ACG..G...C-.T.G..T.A...T........................................................................

MW165662_TA102 ....ACG..G...C-.T.G..TAA...T........................................................................

MW165663_TA103 ....ACG..G...C-.T.G..T.A...T..................................A.....................................

MW165664_TA104 ....ACG..G...C-.T.G..T.A...T...........................................................G............

MW165665_TA105 ....ACG..G..CC-.T.G..T.A...T........................................................................

MW165666_TA106 ....ACG..G...C-.T.G..T.A...T........................................................................

MW165667_TA107 ....ACG..G...C-.T.G..T.A...T........................................................................

MW165668_TA108 ....ACG..G...C-.T.G..T.A...T........................................................................

MW165669_TA109 .T..ACG..G...C-.T.G..T.A...T........................................................................

MW165670_TA110 ....ACG..G...C-.T.G..T.A...T........................................................................

MW165671_TA111 ....ACG..G...C-.T.G..T.A...T........................................................................

MW165672_TA112 ....ACG..G...C-.T.G..T.A...T........................................................................

MW165673_TA113 ....ACG..G...C-.T.G..T.A...T........................................................................

MW165674_TA114 ....ACG..G...C-.T.G..T.A...T.................................T......................................

MW165675_TA115 ....ACG..G...C-.T.G..T.A...T........................................................................

MW165676_TA116 ....ACG..G...C-.T.G..T.A...T........................................................................

MW165677_TA117 ....ACG..G...C-.T.G..T.A...T........................................................................

MW165678_TA118 ....ACG..G...C-.T.G..T.A...T........................................................................

MW165679_TA119 ....ACG..G...C-.T.G..T.A...T.........................T..............................................

MW165680_TA120 ....ACG..G...C-.T.G..T.A...T........................................................................

MW165681_TA121 ....ACG..G...C-.T.G..T.A...T........................................................................

MW165682_TA122 ....ACG..G...C-.T.G..T.A...T........................................................................

MW165683_TA123 ....ACG..G...C-.T.G..T.A...T........................................................................

MW165684_TA124 ....ACG..G...C-.T.G..T.A...T........................................................................

MW165685_TA125 ....ACG..G...C-.T.G..T.A...T............................T...........................................

MW165686_TA126 ....ACG..G...C-.T.G..T.A...T........................................................................

MW165687_TA127 ....ACG..G...C-.T.G..T.A...T........................................................................

MW165688_TA128 ....ACG..G...C-.T.G..T.A...T.........................................................T..............

MW165689_TA129 ....ACG..G...C-.T.G..T.A...T........................................................................

MW165690_TA130 ....ACG..G...C-.T.G..T.A...T.........A..............................................................

MW165691_TA131 ....ACG..G...C-.T.G..T.A...T........................................................................

MW165692_TA132 ....ACG..G...C-.T.G..T.A...T........................................................................

MW165693_TA133 ....ACG..G...C-.T.G..T.A...T........................................................................

MW165694_TA134 ....ACG..G...C-.T.G..T.A...T........................................................................

MW165695_TA135 ....ACG..G...C-.T.G..T.A...T......................T.................................................

MW165696_TA136 ....ACG..G...C-.T.G..T.A...T........................................................................

MW165697_TA137 ....ACG..G...C-.T.G..T.A...T.....................................A..................................

MW165698_TA138 ....ACG..G...C-.T.G..T.A...T........................................................................

MW165699_TA139 ....ACG..G...C-.T.G..T.A...T.........................................C..............................

MW165700_TA140 ....ACG..G...C-.T.G..T.A...T........................................................................

MW165701_TA141 ....ACG..G...C-.T.G..T.A...T........................................................................

MW165702_TA142 ....ACG..G...C-.T.G..T.A...T........................................................................

MW165703_TA143 ....ACG..G...C-.T.G..T.A...T........................................................................

MW165704_TA144 ....ACG..G...C-.T.G..T.A...T........................................................................

MW165705_TA145 ....ACG..G...C-.T.G..T.A...T........................................................................

MW165706_TA146 ....ACG..G...C-.T.G..T.A...T........................................................................

310 320 330 340 350 360 370 380 390 400

....|....|....|....|....|....|....|....|....|....|....|....|....|....|....|....|....|....|....|....|

MW165707_TOR1 GACTTTGGTTCTATTTTGTTGGTTTTAGGTACCAAAGTAATGGTTAATAGGAACAGTTGGGGGCATTCGTATTTAACTGTCAGAGGTGAAATTCTTAGAT

MW165708_TOR2 ....................................................................................................

MW165709_TOR3 ....................................................................................................

MW165710_TOR4 ....................................................................................................

MW165716_TLE1 .............................AG...G.......................................G.........................

MW165561_TA1 ....................................................................................................

MW165562_TA2 ....................................................................................................

MW165563_TA3 ....................................................................................................

MW165564_TA4 ....................................................................................................

MW165565_TA5 ....................................................................................................

MW165566_TA6 ....................................................................................................

MW165567_TA7 ....................................................................................................

MW165568_TA8 ....................................................................................................

MW165569_TA9 ....................................................................................................

MW165570_TA10 ....................................................................................................

MW165571_TA11 ....................................................................................................

MW165572_TA12 ....................................................................................................

MW165573_TA13 ....................................................................................................

MW165574_TA14 ....................................................................................................

MW165575_TA15 ....................................................................................................

MW165576_TA16 ....................................................................................................

MW165577_TA17 ....................................................................................................

MW165578_TA18 ....................................................................................................

MW165579_TA19 ....................................................................................................

MW165580_TA20 ....................................................................................................

MW165581_TA21 ....................................................................................................

MW165582_TA22 ....................................................................................................

MW165583_TA23 ....................................................................................................

MW165584_TA24 ....................................................................................................

MW165585_TA25 ....................................................................................................

MW165586_TA26 ....................................................................................................

MW165587_TA27 ....................................................................................................

MW165588_TA28 ....................................................................................................

MW165589_TA29 ....................................................................................................

MW165590_TA30 ....................................................................................................

MW165591_TA31 ....................................................................................................

MW165592_TA32 ....................................................................................................

MW165593_TA33 ....................................................................................................

MW165594_TA34 ....................................................................................................

MW165595_TA35 ....................................................................................................

MW165596_TA36 ....................................................................................................

MW165597_TA37 ....................................................................................................

MW165598_TA38 ....................................................................................................

MW165599_TA39 ....................................................................................................

MW165600_TA40 ....................................................................................................

MW165601_TA41 ....................................................................................................

MW165602_TA42 ....................................................................................................

MW165603_TA43 ....................................................................................................

MW165604_TA44 ....................................................................................................

MW165605_TA45 ....................................................................................................

MW165606_TA46 ....................................................................................................

MW165607_TA47 ....................................................................................................

MW165608_TA48 ....................................................................................................

MW165609_TA49 ....................................................................................................

MW165610_TA50 ....................................................................................................

MW165611_TA51 ....................................................................................................

MW165612_TA52 ....................................................................................................

MW165613_TA53 ....................................................................................................

MW165614_TA54 ....................................................................................................

MW165615_TA55 ....................................................................................................

MW165616_TA56 ....................................................................................................

MW165617_TA57 ....................................................................................................

MW165618_TA58 ....................................................................................................

MW165619_TA59 ....................................................................................................

MW165620_TA60 ....................................................................................................

MW165621_TA61 ....................................................................................................

MW165622_TA62 ....................................................................................................

MW165623_TA63 ....................................................................................................

MW165624_TA64 ....................................................................................................

MW165625_TA65 ....................................................................................................

MW165626_TA66 ....................................................................................................

MW165627_TA67 ....................................................................................................

MW165628_TA68 ....................................................................................................

MW165629_TA69 ....................................................................................................

MW165630_TA70 ....................................................................................................

MW165631_TA71 ....................................................................................................

MW165632_TA72 ....................................................................................................

MW165633_TA73 ....................................................................................................

MW165634_TA74 ....................................................................................................

MW165635_TA75 ....................................................................................................

MW165636_TA76 ....................................................................................................

MW165637_TA77 ....................................................................................................

MW165638_TA78 ....................................................................................................

MW165639_TA79 ....................................................................................................

MW165640_TA80 .................................G..................................................................

MW165641_TA81 ........................C...........................................................................

MW165642_TA82 ....................................................................................................

MW165643_TA83 ....................................................................................................

MW165644_TA84 ....................................................................................................

MW165645_TA85 ....................................................................................................

MW165646_TA86 ....................................................................................................

MW165647_TA87 .......................................G............................................................

MW165648_TA88 .G..................................................................................................

MW165649_TA89 ....................................................................................................

MW165650_TA90 ....................................................................................................

MW165651_TA91 ....................................................................................................

MW165652_TA92 ....................................................................................................

MW165653_TA93 ....................................................................................................

MW165654_TA94 ....................................................................................................

MW165655_TA95 ....................................................................................................

MW165656_TA96 ....................................................................................................

MW165657_TA97 ............G.......................................................................................

MW165658_TA98 ....................................................................................................

MW165659_TA99 ....................................................................................................

MW165660_TA100 ....................................................................................................

MW165661_TA101 ....................................................................................................

MW165662_TA102 ....................................................................................................

MW165663_TA103 ....................................................................................................

MW165664_TA104 ....................................................................................................

MW165665_TA105 ....................................................................................................

MW165666_TA106 ....................................................................................................

MW165667_TA107 ....................................................................................................

MW165668_TA108 ....................................................................................................

MW165669_TA109 ....................................................................................................

MW165670_TA110 ..............................................................................................A.....

MW165671_TA111 ....................................................................................................

MW165672_TA112 ....................................................................................................

MW165673_TA113 ............................................A.......................................................

MW165674_TA114 ....................................................................................................

MW165675_TA115 ....................................................................................................

MW165676_TA116 ....................................................................................................

MW165677_TA117 ....................................................................................................

MW165678_TA118 ....................................................................................................

MW165679_TA119 ....................................................................................................

MW165680_TA120 ....................................................................A...............................

MW165681_TA121 ....................................................................................................

MW165682_TA122 ..........................................................C.........................................

MW165683_TA123 ....................................................................................................

MW165684_TA124 ....................................................................................................

MW165685_TA125 ....................................................................................................

MW165686_TA126 ....................................................................................................

MW165687_TA127 ....................................................................................................

MW165688_TA128 ....................................................................................................

MW165689_TA129 ....................................................................................................

MW165690_TA130 ....................................................................................................

MW165691_TA131 ....................................................................................................

MW165692_TA132 ..................................................A.................................................

MW165693_TA133 .................................................A..................................................

MW165694_TA134 ....................................................................................................

MW165695_TA135 ....................................................................................................

MW165696_TA136 ....................................................................................................

MW165697_TA137 ....................................................................................................

MW165698_TA138 ....................................................................................................

MW165699_TA139 ....................................................................................................

MW165700_TA140 ....................................................................................................

MW165701_TA141 ..............................G.....................................................................

MW165702_TA142 ....................................................................................................

MW165703_TA143 ....................................................................................................

MW165704_TA144 A...................................................................................................

MW165705_TA145 T...................................................................................................

MW165706_TA146 ....................................................................................................

410 420 430 440 450

....|....|....|....|....|....|....|....|....|....|....|....

MW165707_TOR1 TTGTTAAAGACGAACTACTGCGAAAGCATTTGCCAAGGATGTTTTCATTAATCAAGAAC

MW165708_TOR2 ...........................................................

MW165709_TOR3 ...........................................................

MW165710_TOR4 ...........................................................

MW165716_TLE1 ....C......................................................

MW165561_TA1 ...........................................................

MW165562_TA2 ...........................................................

MW165563_TA3 ...........................................................

MW165564_TA4 ...........................................................

MW165565_TA5 ...........................................................

MW165566_TA6 ...........................................................

MW165567_TA7 ...........................................................

MW165568_TA8 ...........................................................

MW165569_TA9 ...........................................................

MW165570_TA10 ...........................................................

MW165571_TA11 ...........................................................

MW165572_TA12 ...........................................................

MW165573_TA13 ...........................................................

MW165574_TA14 ...........................................................

MW165575_TA15 ...........................................................

MW165576_TA16 ...........................................................

MW165577_TA17 ...........................................................

MW165578_TA18 ...........................................................

MW165579_TA19 ...........................................................

MW165580_TA20 ...........................................................

MW165581_TA21 ...........................................................

MW165582_TA22 ...........................................................

MW165583_TA23 ...........................................................

MW165584_TA24 ...........................................................

MW165585_TA25 ...........................................................

MW165586_TA26 ...........................................................

MW165587_TA27 ...........................................................

MW165588_TA28 .A.........................................................

MW165589_TA29 ...........................................................

MW165590_TA30 ...........................................................

MW165591_TA31 ...........................................................

MW165592_TA32 ...........................................................

MW165593_TA33 ...........................................................

MW165594_TA34 ...........................................................

MW165595_TA35 ...........................................................

MW165596_TA36 ...........................................................

MW165597_TA37 ...........................................................

MW165598_TA38 ...........................................................

MW165599_TA39 ...........................................................

MW165600_TA40 ...........................................................

MW165601_TA41 ...........................................................

MW165602_TA42 ...........................................................

MW165603_TA43 ...........................................................

MW165604_TA44 ...........................................................

MW165605_TA45 ...........................................................

MW165606_TA46 ...........................................................

MW165607_TA47 ...........................................................

MW165608_TA48 ...........................................................

MW165609_TA49 ...........................................................

MW165610_TA50 ...........................................................

MW165611_TA51 ...........................................................

MW165612_TA52 ...........................................................

MW165613_TA53 ...........................................................

MW165614_TA54 ...........................................................

MW165615_TA55 ...........................................................

MW165616_TA56 ...........................................................

MW165617_TA57 ...........................................................

MW165618_TA58 .............................A.............................

MW165619_TA59 .............................C.............................

MW165620_TA60 ...........................................................

MW165621_TA61 ...........................................................

MW165622_TA62 ...........................................................

MW165623_TA63 .....................A.....................................

MW165624_TA64 .....................T.....................................

MW165625_TA65 ...........................................................

MW165626_TA66 ...........................................................

MW165627_TA67 ...........................................................

MW165628_TA68 ...........................................................

MW165629_TA69 ...........................................................

MW165630_TA70 ...........................................................

MW165631_TA71 ...........................................................

MW165632_TA72 ...........................................................

MW165633_TA73 ...........................................................

MW165634_TA74 ...........................................................

MW165635_TA75 ...........................................................

MW165636_TA76 ...........................................................

MW165637_TA77 ...........................................................

MW165638_TA78 ...........................................................

MW165639_TA79 ...........................................................

MW165640_TA80 ...........................................................

MW165641_TA81 ...........................................................

MW165642_TA82 ...........................................................

MW165643_TA83 ...........................................................

MW165644_TA84 ...........................................................

MW165645_TA85 ..C........................................................

MW165646_TA86 ...........................................................

MW165647_TA87 ...........................................................

MW165648_TA88 ...........................................................

MW165649_TA89 ...........................................................

MW165650_TA90 ...........................................................

MW165651_TA91 ...........................................................

MW165652_TA92 ...........................................................

MW165653_TA93 ...........................................................

MW165654_TA94 ...........................................................

MW165655_TA95 ...........................................................

MW165656_TA96 ...........................................................

MW165657_TA97 ...........................................................

MW165658_TA98 ...........................................................

MW165659_TA99 ...........................................................

MW165660_TA100 ...........................................................

MW165661_TA101 ...........................................................

MW165662_TA102 ...........................................................

MW165663_TA103 ...........................................................

MW165664_TA104 ...........................................................

MW165665_TA105 ...........................................................

MW165666_TA106 ...........................................................

MW165667_TA107 ...........................................................

MW165668_TA108 ...........................................................

MW165669_TA109 ...........................................................

MW165670_TA110 ...........................................................

MW165671_TA111 ...........................................................

MW165672_TA112 ...........................................................

MW165673_TA113 ...........................................................

MW165674_TA114 ...........................................................

MW165675_TA115 ...........................................................

MW165676_TA116 ...........................................................

MW165677_TA117 .C.........................................................

MW165678_TA118 ...........................................................

MW165679_TA119 ...........................................................

MW165680_TA120 ...........................................................

MW165681_TA121 ...........................................................

MW165682_TA122 ...........................................................

MW165683_TA123 ...........................................................

MW165684_TA124 ...........................................................

MW165685_TA125 ...........................................................

MW165686_TA126 ...........................................................

MW165687_TA127 ...........................................................

MW165688_TA128 ...........................................................

MW165689_TA129 ...........................................................

MW165690_TA130 ...........................................................

MW165691_TA131 .......................C...................................

MW165692_TA132 ...........................................................

MW165693_TA133 ...........................................................

MW165694_TA134 ...........................................................

MW165695_TA135 ...........................................................

MW165696_TA136 ....................................A......................

MW165697_TA137 ...........................................................

MW165698_TA138 ...........................................................

MW165699_TA139 ...........................................................

MW165700_TA140 ...........................................................

MW165701_TA141 ...........................................................

MW165702_TA142 ...........................................................

MW165703_TA143 ...........................................................

MW165704_TA144 ...........................................................

MW165705_TA145 ...........................................................

MW165706_TA146 ...........................................................

**Figure S2.** Nucleotide alignment of amplicon sequence variants (18S rRNA) of *Theileria* species (*T. orientalis*, TOR1-TOR4; *T. lestoquardi*-like, TLE1; *T. annulata*, TA1-TA146). A dot indicates an identical nucleotide with respect to the top sequence.


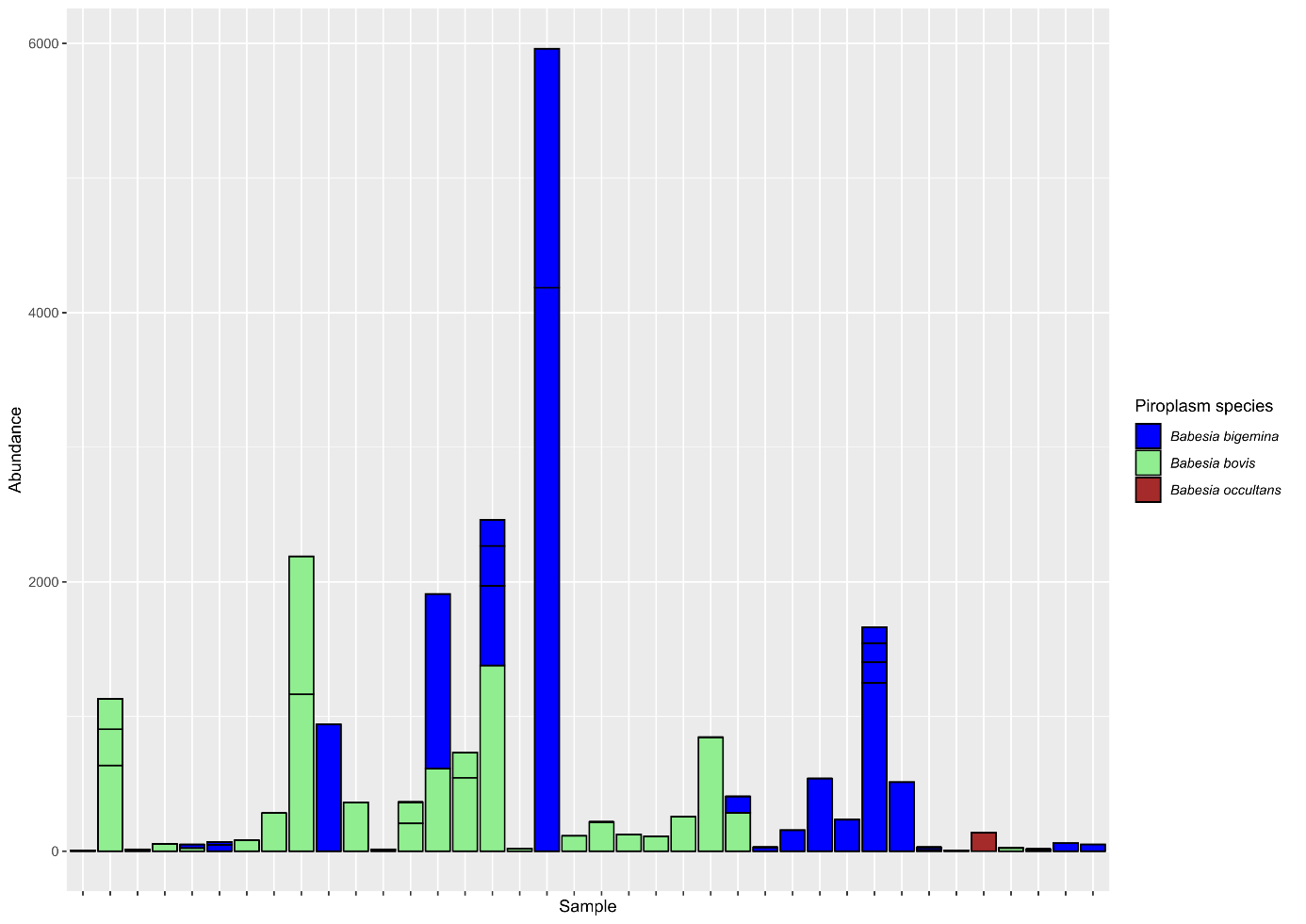


Figure S3. Relative abundance of 18S sequences of Babesia species in individual bovine blood samples from distinct districts in Pakistan.


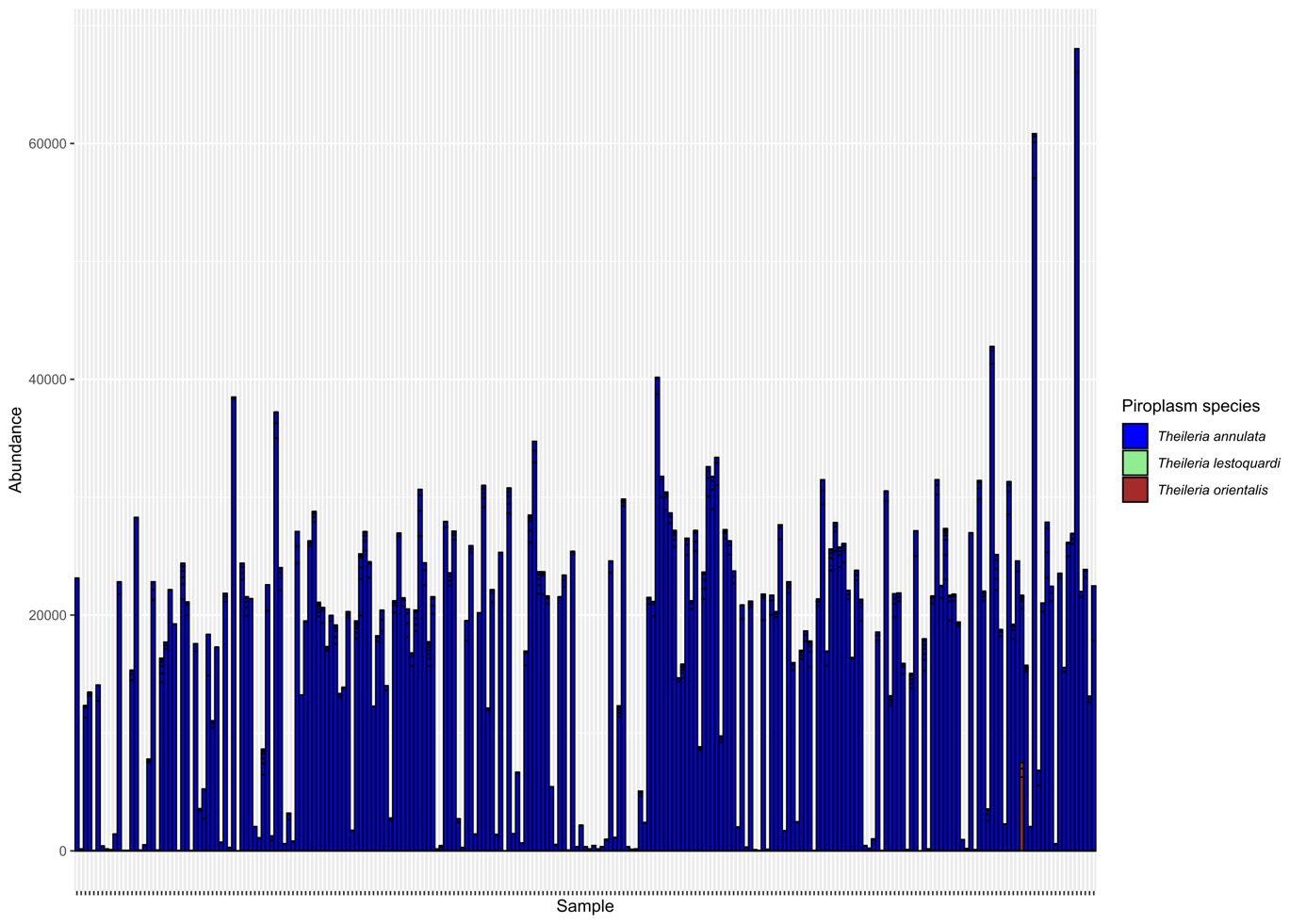


**Figure S4.** Relative abundance of 18S sequences of *Theileria* species in individual bovine blood samples from distinct districts in Pakistan.
